# Cep55 overexpression promotes genomic instability and tumorigenesis in mice

**DOI:** 10.1038/s42003-020-01304-6

**Published:** 2020-10-21

**Authors:** Debottam Sinha, Purba Nag, Devathri Nanayakkara, Pascal H. G. Duijf, Andrew Burgess, Prahlad Raninga, Veronique A. J. Smits, Amanda L. Bain, Goutham Subramanian, Meaghan Wall, John. W. Finnie, Murugan Kalimutho, Kum Kum Khanna

**Affiliations:** 1 QIMR Berghofer Medical Research Institute, 300 Herston Road, Herston, 4006 QLD Australia; 2 School of Environment and Sciences, Griffith University, Nathan, 4111 QLD Australia; 3 University of Queensland Diamantina Institute, The University of Queensland, Translational Research Institute, Brisbane, 4102 QLD Australia; 4 Institute of Health and Biomedical Innovation and School of Biomedical Sciences, Queensland University of Technology, Brisbane, Australia; 5 ANZAC Research Institute, University of Sydney, Sydney, NSW Australia; 6 Unidad de Investigación, Hospital Universitario de Canarias, Tenerife, Spain; 7 Instituto de Tecnologías Biomédicas, Universidad de La Laguna, Tenerife, Spain; 8 Universidad Fernando Pessoa Canarias, Las Palmas de Gran Canaria, Spain; 9 Victorian Cancer Cytogenetics Service, St. Vincent’s Hospital, Fitzroy, Melbourne, Australia; 10 Discipline of Anatomy and Pathology, Adelaide Medical School, University of Adelaide and SA Pathology, Adelaide, Australia; 11 Present Address: Conjoint Internal Medicine Laboratory, Chemical Pathology, Pathology Queensland and Kidney Health Service, Royal Brisbane and Women’s Hospital, Brisbane, 4029 QLD Australia

**Keywords:** Oncogenes, Mechanisms of disease

## Abstract

High expression of centrosomal protein CEP55 has been correlated with clinico-pathological parameters across multiple human cancers. Despite significant in vitro studies and association of aberrantly overexpressed CEP55 with worse prognosis, its causal role in vivo tumorigenesis remains elusive. Here, using a ubiquitously overexpressing transgenic mouse model, we show that *Cep55* overexpression causes spontaneous tumorigenesis and accelerates *Trp53*^*+/−*^ induced tumours in vivo. At the cellular level, using mouse embryonic fibroblasts (MEFs), we demonstrate that *Cep55* overexpression induces proliferation advantage by modulating multiple cellular signalling networks including the hyperactivation of the Pi3k/Akt pathway. Notably, *Cep55* overexpressing MEFs have a compromised Chk1-dependent S-phase checkpoint, causing increased replication speed and DNA damage, resulting in a prolonged aberrant mitotic division. Importantly, this phenotype was rescued by pharmacological inhibition of Pi3k/Akt or expression of mutant Chk1 (S280A) protein, which is insensitive to regulation by active Akt, in *Cep55* overexpressing MEFs. Moreover, we report that *Cep55* overexpression causes stabilized microtubules. Collectively, our data demonstrates causative effects of deregulated Cep55 on genome stability and tumorigenesis which have potential implications for tumour initiation and therapy development.

## Abstract (web summary)

Sinha et al. demonstrate that overexpression of centrosomal protein Cep55 in mice is sufficient to cause a wide-spectrum of cancer via multiple mechanisms including hyperactivation of the Pi3k/Akt pathway, stabilized microtubules and a defective replication checkpoint response. These findings are relevant to human cancers as high CEP55 expression is associated with worse prognosis across multiple cancer types.

## Introduction

Genomic instability (GI) is a hallmark of almost all human cancers. Chromosomal instability (CIN) is a major form of GI, which refers to the acquisition of abnormal chromosome numbers or structures^1^. CIN in cancers primarily occurs due to defective mitosis, including biased chromosome segregation and failure to undergo cytokinesis. Both mitotic checkpoint weakness and/or hyperactivation can also lead to CIN, exploring its genetic basis has the potential to uncover major mechanism of GI in cancers and therapeutic modality^2^.

CEP55 is a coiled-coil centrosomal protein which plays a critical role in cytokinetic abscission during mitotic exit^3^. CEP55 is a cancer testis antigen expressed during embryogenesis and is silent in most adult tissues except testis; however, it is re-expressed in a wide variety of cancers^4^. Over the last decade, multiple studies have shown variable associations of overexpressed CEP55 with poor prognosis in human cancers (reviewed by Jeffery et al.^4^). On the other hand, loss-of-function mutations in *CEP55* cause late gestation lethality and Meckel-like and MARCH syndromes^5–8^. Notably, increased CEP55 expression correlates with functional aneuploidy in multiple cancer types, as defined by the *CIN70* gene signature^9^. It is also part of a 10-gene signature associated with drug resistance, CIN, and cell proliferation^10^. Moreover, as part of the 31-gene cell-cycle progression (CCP) signature, it strongly correlates with actively proliferating prostate cancer cells^11^. Likewise, we have shown that *CEP55* is part of a 206 gene signature, representing genes enriched in promoting CIN, associated with aggressiveness of triple-negative breast cancer (TNBC)^12^.

Mechanistically, wild-type *TP53* suppresses CEP55 through PLK1 downregulation and therefore, cancers with *TP53* mutations often have elevated CEP55 levels^13^. In human cancers, CEP55-overexpression results in cell transformation, proliferation, epithelial-to-mesenchymal transition, invasion, and cell migration via upregulation of the PI3K/AKT pathway through direct interaction with the p110 catalytic subunit of PI3K^14,15^. Likewise, CEP55 interacts with JAK2 kinase and promotes its phosphorylation^16^. We have recently shown that *Cep55* overexpression in mice causes male-specific sterility through the hyperactivation of Pi3k/Akt pathway in mice^17^. Furthermore, we showed that CEP55 is a determinant of aneuploid cell fate during perturbed mitosis in breast cancers and could be targeted through MEK1/2-PLK1 inhibition^18^. Moreover, recently *Cep55* has been shown to regulate anaphase I of the meiotic oocytes^19^. Collectively, these studies highlight the association of CEP55 overexpression with various human malignancies in a context-dependent manner. Though these in vitro and clinical correlation studies have so far established the link between CEP55 overexpression and cancer, the underlying mechanism by which CEP55 promotes tumorigenesis in vivo remains elusive.

Here, we report that *Cep55* overexpression in a mouse model causes high incidence of spontaneous tumorigenesis with a wide spectrum of highly proliferative and metastatic tumors. Notably, *Cep55* overexpression accelerates *Trp53*^*+/−*^-induced tumorigenesis. Using mouse embryonic fibroblasts (MEFs), we show that *Cep55* overexpression facilitates rapid proliferation by modulating multiple cell signaling networks, particularly hyperactivation of Pi3k/Akt pathway which consequently impacts on Chk1-dependent replication checkpoint. Moreover, we found that *Cep55* overexpression causes both numerical and structural CIN due to stabilized microtubules. Collectively, our data demonstrate a causal link of overexpressed Cep55 with tumorigenesis, driven through its multiple cellular functions.

## Results

### Cep55 overexpression drives tumorigenesis in vivo

**Fig. 1:**
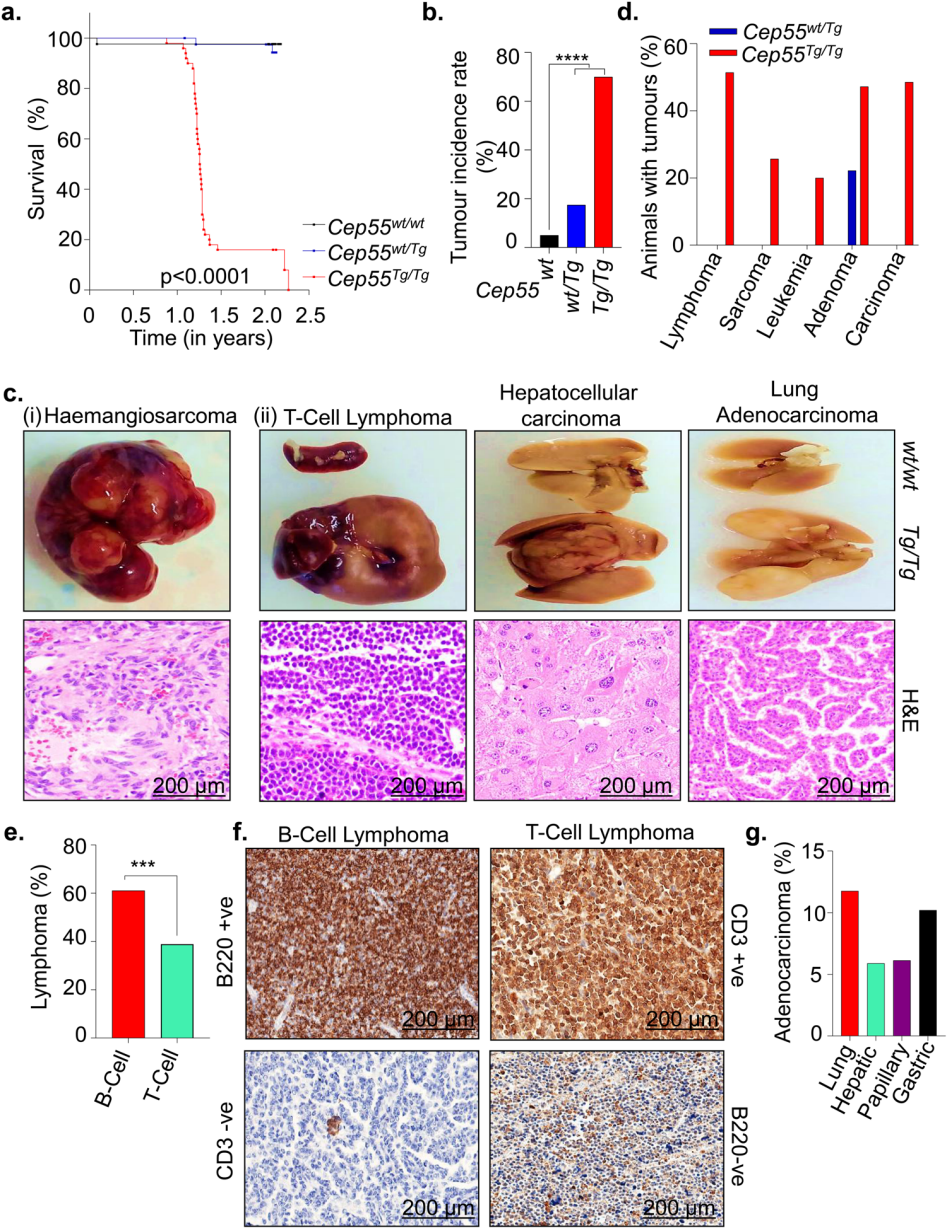
Cep55 overexpression causes spontaneous tumorigenesis in vivo. **a** Kaplan–Meier survival analysis of mice of indicated genotypes (*n* ≥ 40 per group) showing that *Cep55*^*Tg/Tg*^ mice were more susceptible to form tumors compared to their control counterparts; Log-rank (Mantel–Cox) test was performed to determine *P-value* < 0.0001. **b** Percentage of cancer incidence rate among mice of indicated genotypes (*n* ≥ 40 per group); Fischer exact test was performed to determine *P-value* < 0.00001 (****). **c** Representation images of gross morphology (upper panels) and H&E stained microscopic images (lower panels) of selected sections of (i) haemangiosarcoma in liver of tumor-bearing *Cep55*^*Tg/Tg*^ mice from which the tumor cell lines (TCL) were established (discussed later in Supplementary Fig. 4) (ii) other tumor lesions (T-cell lymphoma, hepatocellular carcinoma, and Lung Adenocarcinoma) from different organs among *Cep55*^*Tg/Tg*^ mice (scale bars, 200 µm). **d** Percentage of animals with respective cancer types observed in the transgenic cohorts. **e** Percentage of animal with types of lymphomas observed in the respective tumor-bearing *Cep55*^*Tg/Tg*^ mice. Fischer exact test was performed to determine *P*-value < 0.0029 (***). **f** Representative images of B220 and CD3 immunostaining used to categorize the respective types of lymphomas. B220+ve and CD3-ve were classified as B-cell lymphoma while CD3+ve and B220-ve were classified as T-Cell lymphoma (scale bars, 200 µm). **g** Percentage of adenocarcinoma in the respective organs observed in the tumors bearing *Cep55*^*Tg/Tg*^ mice.

To characterize the pathophysiological role of CEP55 overexpression in vivo, we utilized our recently reported transgenic mouse model^17^. Since *CEP55* is highly overexpressed in multiple human cancers irrespective of its role in cell division (Supplementary Fig. 1A–E), we asked if *Cep55* overexpression causes spontaneous tumorigenesis in vivo. We monitored a cohort of wild type (herein referred to as *Cep55*^*wt/wt*^, *n* = 40), heterozygous transgenic (*Cep55*^*wt/Tg*^, *n* = 40), and homozygous transgenic (*Cep55*^*Tg/Tg*^, *n* = 50) *Cep55* mice (both males and females) over a period of 2.5 years for spontaneous tumor formation. We observed that the *Cep55*^*Tg/Tg*^ mice developed various types of tumors at relatively long latencies (median survival 15 months) (Table 1) compared to other well-known oncogenic tumor models (*K-ras*^*G12D*^ ^20^, *Pten*^*+/−*^ ^21^, and *Trp53*^−*/−*22,23^). However, homozygous-*Cep55* overexpressing mice succumbed to cancer significantly earlier (*p* < 0.0001) than *Cep55*^*wt/Tg*^ and *Cep55*^*wt/wt*^ littermates (Fig. 1a). Notably, more than 50% of the *Cep55*^*Tg/Tg*^ mice were culled between 13 and 15 months due to irreversible weight loss (>15%), reluctance to move and/or eat and showed development of tumors (Supplementary Fig. 2A).

**Table 1:** Distribution of cancer spectrum in Cep55 transgenic mice.

No.	Cancerous Lesions	*Cep55*^*wt/wt*^(*n* = 40)		*Cep55*^*wt/Tg*^(*n* = 40)		*Cep55*^*Tg/Tg*^(*n* = 50)		*Cep55*^*wt/wt*^ vs *Cep55*^*Tg/Tg*^*, Cep55*^*wt/Tg*^ vs *Cep55*^*Tg/Tg*^	
		#	%	#	%	#	%	*p* values^a^	
1	Lymphoma	0	0	0	0	18	51.42	6.0 × 10^−6^	6.0 × 10^−6^
	B-Cell Lymphoma	–		–		11	61.11	0.0010	0.0010
	T-Cell Lymphoma	–		–		7	38.88	0.0159	0.0159
2	Undifferentiated sarcoma	0	0	0	0	9	25.71	0.0039	0.0039
	Fibrosarcoma	–		–		3	33.33	0.2509	0.2509
	Hemangiosarcoma	–		–		6	66.67	0.0317	0.0317
3	Bronchogenic adenocarcinoma	0	0	0	0	6	17.14	0.0317	0.0317
4	Hepatocellular Carcinoma	0	0	0	0	3	8.57	0.2509	0.2509
5	Gastric Carcinoma	0	0	0	0	5	14.28	0.0632	0.0632
6	Intestinal Papillary Carcinoma	0	0	0	0	3	8.57	0.2509	0.2509
7	Myelogenous Leukemia	0	0	0	0	7	20	0.0159	0.0159
8	Hepatic Hyperplasia (foci of cellular alteration)	1	2.22	4	8.69	12	34.28	0.0051	0.1019
9	Splenic follicular Hyperplasia	1	2.22	4	8.69	8	66.66	0.0398	0.5373
10	Endometrial Hyperplasia	0	0	0	0	4	33.33	0.1259	0.1259
11	Lipoma	0	0	0	0	1	2.9	1.0	1.0
12	Alveolar-Bronchiolar Adenoma	0	0	10	22.22	15	42.85	0.0001	0.6426
13	Hepatoma	0	0	2	4.34	2	5.7	0.5006	1.0

^a^*P* values: Fisher’s exact tests.

We observed that 70% (35/50) of the *Cep55*^*Tg/Tg*^ mice developed a wide spectrum of tumor lesions, including lymphoma, sarcoma, leukemia, and various adenocarcinomas (Fisher exact test *p* < 0.00001; Fig. 1b–d, Supplementary Fig. 2B and Table 1) compared to only 17.5% (7/40) in *Cep55*^*wt/Tg*^ and 5% (2/40) in *Cep55*^*wt/wt*^ littermates (Fig. 1b). Notably, the tumor burden observed in *Cep55*^*Tg/Tg*^ mice varied between 1 and 3 tumors per animal (Supplementary Fig. 2C) with tumors originating in multiple tissue types (Supplementary Fig. 2D) in comparison to *Cep55*^*wt/Tg*^, which uniformly developed only adenomas in the lung. Likewise, the *Cep55*^*Tg/Tg*^ mice also exhibited a higher incidence of lymphomas, in particular more B-cell lymphoma (1.5-fold) than the T-cell lymphoma (Fig. 1d, e, Table 1). Immunohistochemistry (IHC) staining using B220 (B-cell marker) and CD3 (T-cell marker) specified the incidence of B-cell and T-cell lymphomas, respectively (Fisher exact test *p* < 0.0029; Fig. 1e, f). Independently, we observed a higher incidence of sarcomas, particularly haemangiosarcoma than fibrosarcoma (in liver and spleen) (Supplementary Fig. 2D, E) and a higher incidence of lung and gastric adenocarcinomas compared to other carcinomas (Fig. 1g). We also observed a significant increase in hyperplastic lesions (in liver, spleen, and endometrium) in *Cep55*^*Tg/Tg*^ mice compared to the cohort of other genotypes (Fisher exact test *p* < 0.0001; Supplementary Fig. 2F).

The primary cancers observed in the *Cep55*^*Tg/Tg*^ mice were highly aggressive in nature with increased Ki67 positivity staining compared to adjacent tissues, as perceived by the gross morphology and mass of the organs in which these tumors originated (Fig. 1c, Supplementary Fig. 2G, H). In addition, we observed that ~16% of the mice developed metastases in the lungs and liver (Supplementary Fig. 2I). Collectively, these data highlight that *Cep55* overexpression alone is sufficient to drive tumorigenesis in mice, causing a broad spectrum of cancers and associated with metastasis.

### Cep55 overexpression accelerates *Trp53*^+/−^ induced tumor development in mice

**Fig. 2:**
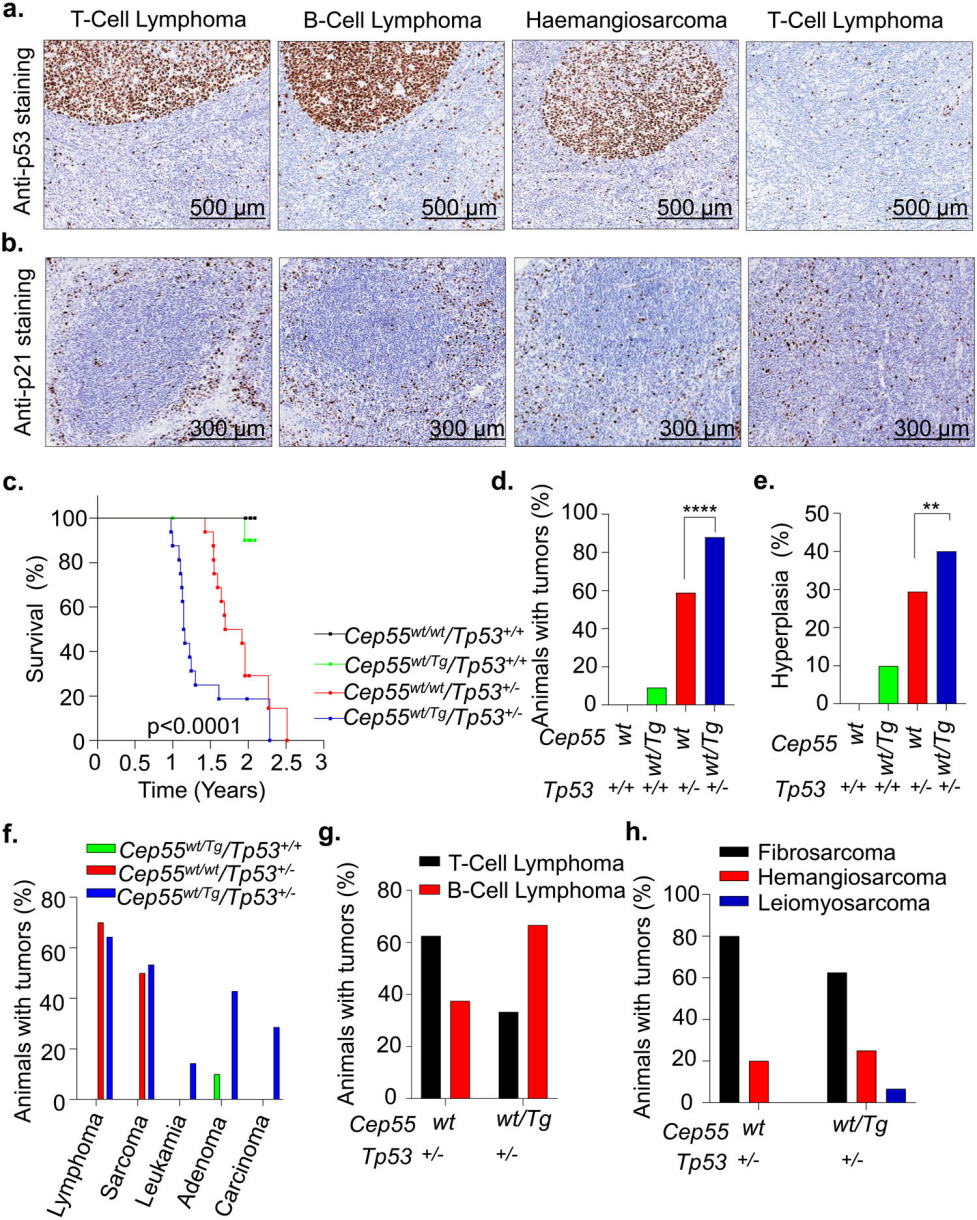
Heterozygous Cep55 transgenic expression accelerates *Trp53*^+/−^-induced tumorigenesis in mice. **a** Representative images of p53 immunohistochemical staining on tumor sections of respective subtypes observed in the *Cep55*^*Tg/Tg*^ mice showing the presence of p53 positive cells in the tumor lesion compared to adjacent normal tissue from the same mice (scale bars, 500 µm). **b** Representative images of p21 immunohistochemical staining on tumor sections of respective subtypes observed in the *Cep55*^*Tg/Tg*^ mice showing the presence of p21 negative cells in the tumor lesion compared to adjacent normal tissue from the same mice (scale bars, 300 µm). **c** Kaplan–Meier survival analysis highlighting the tumor-free survival of the mice of indicated genotypes (*n* ≥ 10 per group) demonstrating that *Cep55*^*wt/Tg*^*; Trp53*^*wt/−*^ mice were more susceptible to form tumors with a shorter latency period (~14 months) compared to control littermates; Log-rank (Mantel–Cox) test was performed to determine *P-value* < 0.0001. **d**–**h** Percentages of overall cancer incidence (**d**), hyperplastic lesions (**e**), cancer spectrum (**f**), lymphoma (**g**) and sarcoma burden (**h**) among mice of indicated genotypes (*n* ≥ 10 per group). Fischer exact test was performed to calculate *P-value* < 0.01 (**) and <0.0001 (****).

Our data suggest that *Cep55* overexpression-induced tumorigenesis mimics the tumorigenesis pattern observed in *Trp53*^*−/−*^ mice^22,23^, as it induces a significantly higher percentage of lymphomas (~51%) and sarcomas (~25%) (Fig. 1d). A previous report has shown that wild-type TP53 restrains CEP55 expression through PLK1^13^. In addition, data mining suggests that *CEP55* levels are significantly higher in lung and hepatocellular tumors that exhibit allelic *TP53* copy number loss than in *TP53* diploid tumors (both p < 0.0001, Mann-Whitney *U* test) (Supplementary Fig. 3A). Consistent with this, we observed a high p53 protein level, which is most likely an indication of mutated *Trp53*, as well as reduced staining of its target p21 in representative *Cep55*^*Tg/Tg*^ tumor tissues than normal adjacent tissues (Fig. 2a, b)

Next, we inter-crossed *Cep55*^*Tg/Tg*^ female mice with *Trp53*^*−/−*^ male mice to establish bi-transgenic cohorts of *Cep55*^*wt/Tg*^*;Trp53*^*+/−*^ (*n* = 15), *Cep55*^*wt/wt*^*;Trp53*^*+/−*^ (*n* = 17), *Cep55*^*wt/Tg*^*;Trp53*^*+/+*^ (*n* = 11), and *Cep55*^*wt/wt*^*;Trp53*^*+/+*^ (*n* = 10) mice. These cohorts of mice were monitored regularly for a period of 2.5 years for spontaneous tumor development. Interestingly, we observed that the *Cep55*^*wt/Tg*^*;Trp53*^*+/−*^ mice succumbed to a broad spectrum of cancer development (spleen, liver, and lung) with reduced latency (median survival of 13.8 months; *p* < 0.0001) when compared to the *Cep55*^*wt/wt*^;*Trp53*^*+/−*^ cohort (median survival of 21.6 months) (Fig. 2c, Supplementary Fig. 3B–F, Supplementary Table 1). The histological features observed across these tumors are described in Supplementary Table 2. Notably, the entire cohort of *Cep55*^wt/Tg^;*Trp53*^+/−^ mice exhibited a time frame of tumor development similar to that of *Cep55*^*Tg/Tg*^ mice (Fig. 2c).

Further, the incidence of tumorigenesis observed in *Cep55*^*wt/Tg*^*;Trp53*^*+/−*^ mice was also significantly higher (~85%; Fisher exact test *p* < 0.0001) in comparison to *Cep55*^*wt/wt*^*;Trp53*^*wt/−*^ (~50%) with 1–3 tumors per animal (Fig. 2d). The *Cep55*^*wt/Tg*^*;Trp53*^*+/−*^ mice also displayed a significantly higher incidence of hyperplastic lesions (Fisher exact test *p* < 0.01) (Fig. 2e), and a similar incidence to that observed in *Cep55*^*Tg/Tg*^ mice (Supplementary Fig. 2F). Histopathological analysis indicated the presence of a number of neoplastic lesions (Fig. 2f, Supplementary Fig. 3B, Supplementary Table 1) that were similarly observed in *Cep55*^*Tg/Tg*^ mice (Fig. 1c, d, and Table 1). Notably, though similar fractions of *Cep55*^*wt/wt*^*; Trp53*^*+/−*^ and *Cep55*^*wt/Tg*^*; Trp53*^*+/−*^ animals developed lymphomas and sarcomas (Fig. 2f); however, their lymphoma spectrums were different. There was a higher incidence of B-cell lymphomas than T-cell lymphomas in the *Cep55*^*wt/Tg*^*;Trp53*^*+/−*^ mice compared to *Cep55*^*wt/wt*^;*Trp53*^*+/−*^ mice (Fig. 2g). Further, the *Cep55*^*wt/Tg*^*;Trp53*^*+/−*^ mice demonstrate a similar occurrence of fibrosarcoma and haemangiosarcoma (in liver and spleen), as observed in *Cep55*^*Tg/Tg*^ mice (Fig. 2h). Taken together, this suggests that Cep55 overexpressing tissues have better tumor incidence when p53 protective effect is reduced in p53-heterozygous compared to p53-wild-type animals.

### Cep55 overexpression confers a survival advantage through activation of signaling networks

**Fig. 3:**
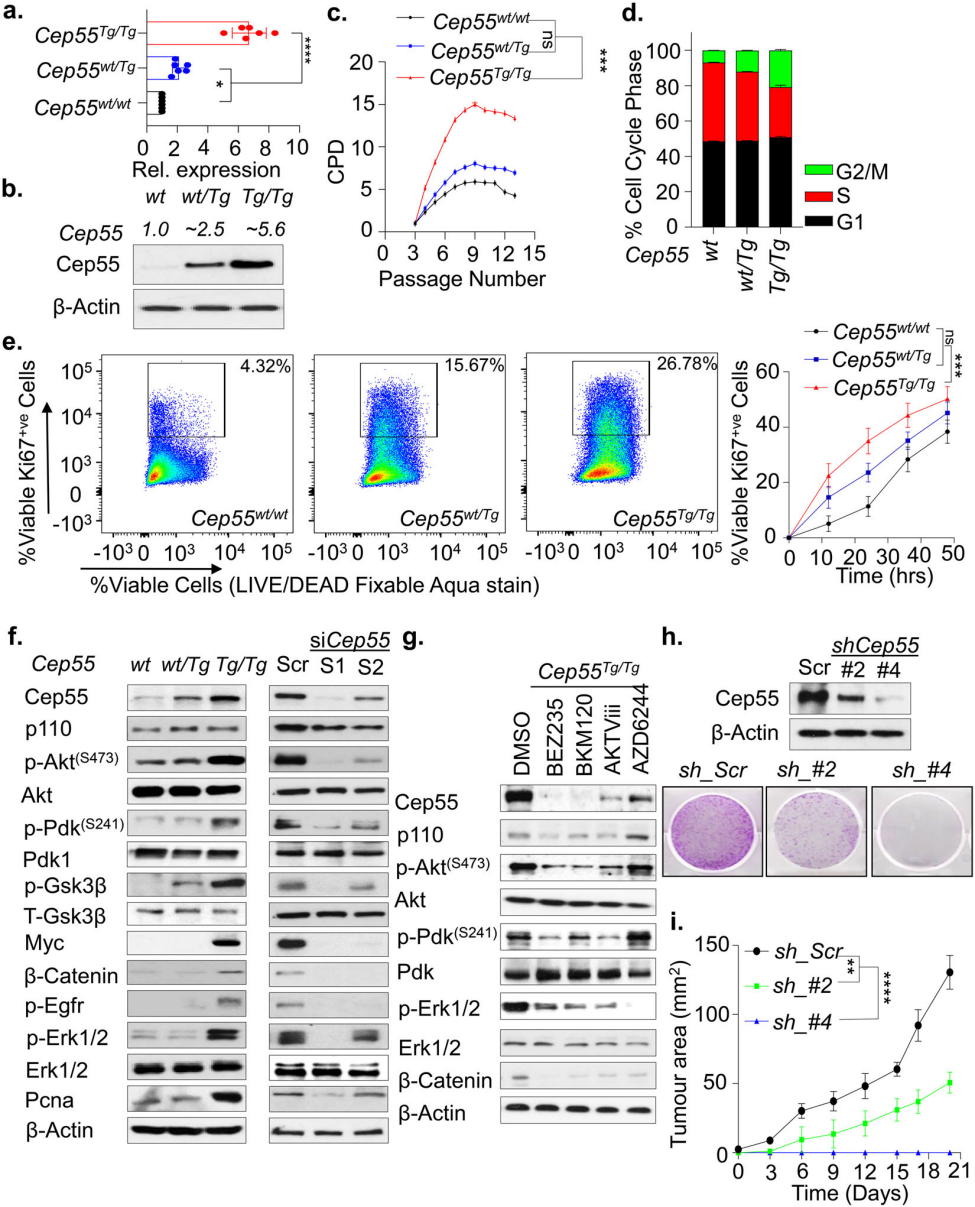
Cep55 confers survival advantage through activation of signaling networks. **a** Expression of Cep55 transcripts observed in the primary mouse embryonic fibroblasts (MEFs) of respective transgenic Cep55 genotypes. Three independent experiments with two technical replicates were performed. Error bars represent ± Standard Deviation (SD). One-way ANOVA test was performed to determine *P-value* < 0.05 (*) and < 0.0001 (****). **b** Immunoblot analysis of Cep55 expression in the whole cell lysates of the primary MEFs of each genotype. β-Actin was used as a loading control and relative fold difference in expression of Cep55, (indicated above calculated by densitometric analysis using ImageJ) observed among the MEFs of respective genotype (number of experimental representation, *n* = 2). **c** Proliferation measured as a function of passage number [indicated as CPD (cumulative population density)] using NIH-3T3 protocol in primary *Cep55*^*Tg/Tg*^ MEFs in comparison to its littermates (*n* = 3 independent experiments with two technical replicates were performed. Error bars represent ± SD). One-way ANOVA test was performed to determine *P-value* < 0.0001 (****). **d** Cell-cycle profile of primary MEFs of indicated genotype measured post 24 h of culture by propidium iodide staining followed by FACS (*n* = 3 independent experiments with two technical replicates). Error bars represent ± SD). Two-way ANOVA test was performed to determine *P-value* is demonstrated in Supplementary Table 3. **e** FACS plot representing the percentage of Ki67 positive staining of viable cells from the respective genotype post 12 h of culture wherein 100,000 viable events were collected for each genotype (left). Quantification of the percentage of Ki67 positive viable cells of each genotype at the representative time points (right). Error bars represent the ± SD from three independent experiments. One-way ANOVA test was performed to determine *P-value;* <0.001 (***) and ns (not significant). **f** Immunoblot analysis of the whole cell lysates collected post 24 h culture from the immortalized MEF’s of indicated genotypes (left panel) and post 48 h from the respective siRNA treated *Cep55*^*Tg/Tg*^ MEFs (right panel) indicating the impact of Cep55 overexpression on multiple cell signaling pathways. β-Actin was used as a loading control. **g** Immunoblot analysis of the whole cell lysates collected after 24 h of treatment of immortalized *Cep55*^*Tg/Tg*^ MEFs with the respecting inhibitors such as BEZ235 (pan-PI3K/AKT inhibitor), BKM120 (pan-PI3K inhibitor), AKTVIII (AKT inhibitor), and AZD6244 (MEK1/2 inhibitor). β-Actin was used as a loading control. **h** Immunoblot analysis of the whole-cell lysates collected from the respective isogenic *Cep55*-depleted TCLs at 24 h validating the levels of Cep55 expression. β-Actin was used as a loading control (top panel). Representative images of colony formation at 14 days determined using crystal violet staining in control and *Cep55*-depleted TCLs (bottom panel). **i** Six-week-old female NOD/Scid cohorts of mice were injected subcutaneously with the control and *Cep55*-depleted clones. Growth rates (area, mm^2^) of the tumors were measured using a digital caliper. Differences in growth were determined using Student’s *t* test, *P* ≤ 0.0001 (****). Graph represents the mean tumor area ± SD, *n* = 5 mice/group.

In multiple human cancers, deregulated expression of CEP55 has been linked to enhanced proliferation, migration, invasion, epithelial-mesenchymal transition, and tumorigenesis^4^. To analyze the impact of *Cep55* overexpression in vitro, we use primary and spontaneously immortalized MEFs isolated from E13.5 embryos (Supplementary Fig. 4A). We observed significantly higher *Cep55* transcript and protein levels in the primary *Cep55*^*Tg/Tg*^ MEFs compared to MEFs from other genotypes (Fig. 3a, b). Next, to determine the growth potential and the senescence rate in the primary MEFs, we performed a 3T3 assay and observed that the *Cep55*^*Tg/Tg*^ primary MEFs had a significantly higher growth rate along with more G2/M cells compared to *Cep55*^*wt/Tg*^ and *Cep55*^*wt/wt*^ MEFs (Fig. 3c, d). Likewise, the immortalized *Cep55*^*Tg/Tg*^ MEFs also exhibited similar enhanced proliferative capacity and increased Ki67 staining over time (Fig. 3e and Supplementary Fig. 4C). To define if *Cep55* overexpression alone could confer enhanced proliferative capacity independent of mitogenic signals, we serum-starved the immortalized MEFs of each genotypes and observed higher cell proliferation capacity in *Cep55*^*Tg/Tg*^ MEFs (~60 h) compared to MEFs from other genotypes, highlighting a self-mitogen gaining capability to proliferate and survive in conditions of serum-starvation (Supplementary Fig. 4D).

CEP55 has been shown to upregulate AKT phosphorylation through direct interaction with p110 catalytic subunit of PI3 kinase (PI3K) and enhance cell proliferation in vitro^14,15,17^. Likewise, we have shown that MYC regulates CEP55 transcriptionally in breast cancer^18^. Thus, to characterize the molecular signaling involved in cell proliferation and survival, we investigated the impact of *Cep55* overexpression on Pi3k/Akt - and Erk-dependent signaling networks. Interestingly, immunoblot analysis using whole cell lysates from the MEFs of each genotype demonstrated *Cep55* dosage-dependent increase in phosphorylation of Akt^S473^ and its upstream regulator Pdk1^S241^ in *Cep55*^*Tg/Tg*^ MEFs compared to wild type and heterozygous MEFs (Fig. 3f). In addition, we also observed an upregulation of Mapk-dependent signaling molecules, including increased-phosphorylation of Egfr, Erk1/2, Myc, and β-catenin, along with increased Pcna, a proliferation marker, in *Cep55*^*Tg/Tg*^ MEFs (Fig. 3f). Similar changes were observed in representative tissue lysates (Supplementary Fig. 4E). Notably, the effects on the signaling networks were specific to *Cep55* overexpression as knockdown of *Cep55* using two different siRNA oligonucleotides in *Cep55*^*Tg/Tg*^ MEFs remarkably diminished Pi3k/Akt and Mapk-dependent signaling pathway activities (Fig. 3f). Reconstitution of Cep55 in siRNA knockdown MEFs rescued the signaling networks of Pi3k/Akt and Mapk (Supplementary Fig. 4F). Furthermore, to characterize the role of *Cep55* overexpression in promoting cell proliferation and survival through activated signaling pathways, we used a wide range of Pi3k/Akt, mTor and Erk1/2 pathway-specific inhibitors. We observed that the *Cep55*^*Tg/Tg*^ MEFs were significantly more sensitive to Akt, Pi3K and pan-Pi3k/Akt/mTor inhibitors, but not to mTor or Erk1/2 inhibitor treatments alone (Supplementary Fig. 4G). Blocking some of these signaling pathways but not others markedly reduced Cep55 levels suggesting positive feedback loops between Cep55 and these signaling pathways (Fig. 3g and Supplementary Fig. 4H)).

To further decipher the impact of overexpressed *Cep55* on tumorigenesis, we established cell lines from some of the tumors that developed in *Cep55* overexpressing mice (herein abbreviated as tumor cell lines (TCLs)), in particular haemangiosarcoma of the liver (Fig. 1ci). These cells exhibited a mixed population of aneuploidy (both bi- and multinucleated), implying a genomically unstable phenotype (Supplementary Fig. 5A). Similarly, upon transient *Cep55* knockdown using siRNA in the TCL, these cells significantly grew slower than siscramble transfected cells with a concomitant reduction in signaling networks that were complemented after the restoration of Cep55 expression (Supplementary Fig. 5B, C). Likewise, constitutive *Cep55* knockdown in this line using shRNAs reduced anchorage-independent colony formation, G2/M cell population along with reduced proliferation capacity and tumor formation dependent on the extent of reduction of Cep55 levels (Fig. 3h, i, Supplementary Fig. 5D–G). Consistently, *Cep55* knockdown TCL was significantly refractory to Pi3k/Akt inhibitor sensitivity (Supplementary Fig. 5H), suggesting a dependency on Pi3k/Akt signaling. Taken together, these data highlight the crucial role of *Cep55* in regulating proliferation and survival-associated signaling networks and its essential function in tumor formation.

### Cep55 overexpression leads to altered Chk1 distribution causing replication stress in an Akt-dependent manner

**Fig. 4:**
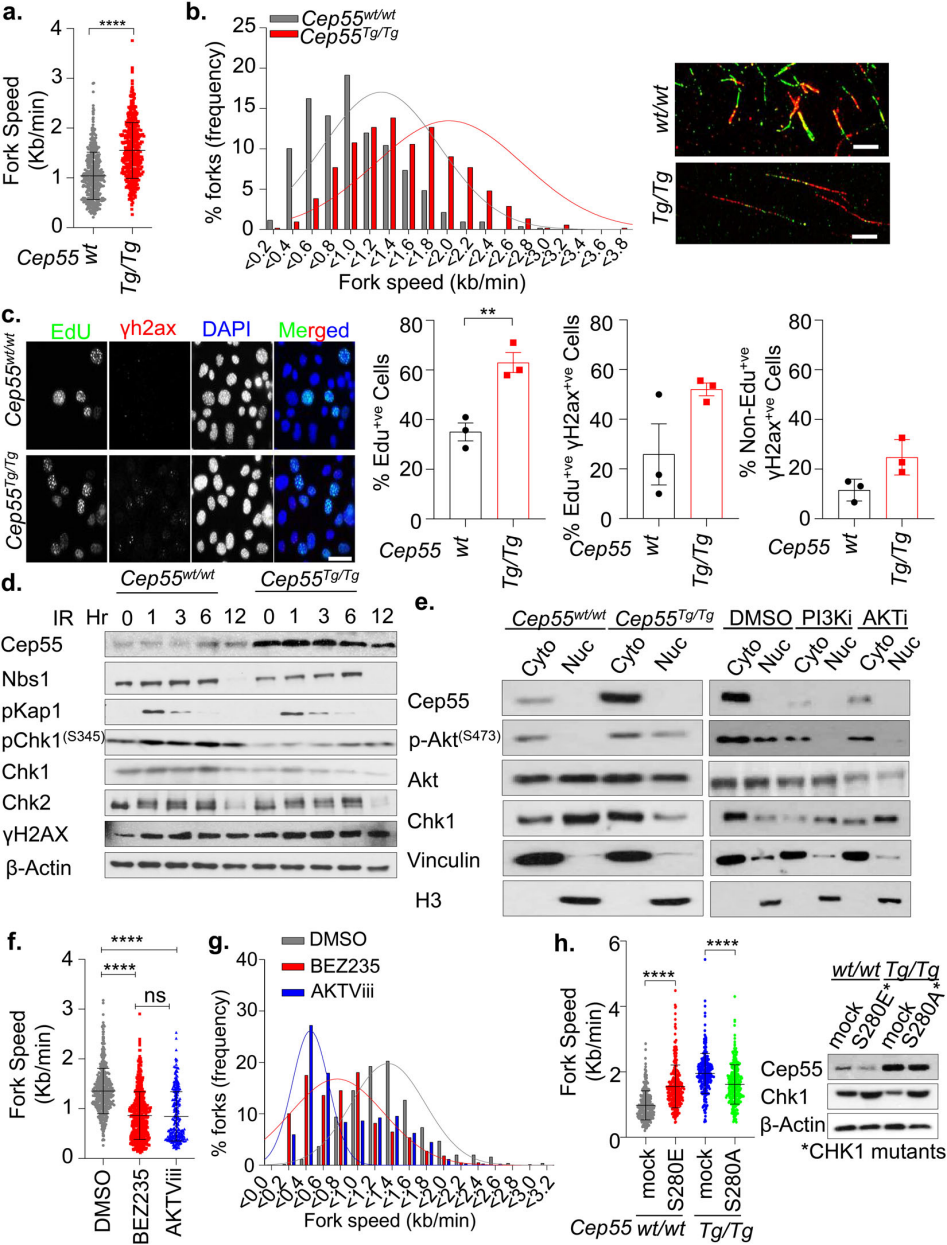
Cep55 overexpression causes replication stress. **a**, **b** Statistical representation of velocity of progressing forks (**a**) and frequency distribution of fork speeds (**b**) was determined using DNA fiber analysis. Indicated immortalized MEFs were pulsed labeled with CldU (green) and IdU (red) for 20 min each and the fibers were imaged and quantified. Representative images of respective genotypes are shown on the right-hand panel. More than 500 fibers from each genotype were analyzed from two independent experiments with error bars in A representing the ±SD. Unpaired *t* test with and without Welch’s correction between two groups was used to determine the statistical *P-value*; < 0.0001 (****). **c** Representative images of immunofluorescence of EdU (S-phase cells) positivity (green) allowed to label for an hour alongside double-stranded DNA breaks marker γ-H2ax (red) observed in the immortalized MEFs (*n* = 150 cells were counted per experiment) of indicated genotypes are shown on the left-hand panel. DNA was marked using DAPI (blue). The statistical representation of the percentages of EdU positive cells; γ-H2ax in EdU positive or negative cells are demonstrated in the right-hand side panel. Error bars represent the ± SD from three independent experiments. Unpaired *t* test was performed to determine *P-value* < 0.0067 (**). **d** Immunoblot analysis of DNA damage response proteins from indicated immortalized MEFs after challenged with 6-Gy irradiation. β-Actin was used as loading control. **e** Immunoblot analysis of cytoplasmic-nuclear fractionation was performed using the indicated immortalized MEFs to determined Chk1 protein distributions with and without inhibitor treatments. Cells were treated for 6 h with inhibitors prior to the assay. H3 and Vincullin were used as fractionation loading controls for nuclear and cytoplasmic fractions, respectively. **f** Quantification of replication fork speed observed in the immortalized *Cep55*^*Tg/Tg*^ MEFs after being challenged with BEZ235 (pan-PI3K/AKT signaling pathways inhibitor), and AKTViii (was used to inhibit Akt signaling). *Cep55*^*Tg/Tg*^ MEFs were pretreated for 6 h with indicated inhibitors and forks speeds were determined. At least 300 fibers from each genotype were analyzed from two independent experiments with error bars representing ±SD. One-way ANOVA with Brown-Forsythe test was used to determine *P-value;* < 0.0001 (****) and ns (not significant). **g** Distributions of replication fork speeds frequency from panel **f**. **h** Statistical representation of velocity of progressing forks as indicated in (**a**). Both cell lines were transiently transfected with 1.5 µg of indicated mutant constructs (*CHK1* mutants -*S280A* and *S280E,* respectively, shown by western blotting (right)) for 24 h and DNA fiber analysis was performed. At least 300 fibers from each genotype were analyzed from three independent experiments with error bars representing ±SD. One-way ANOVA with Brown–Forsythe test was used to determine *P-value* < 0.0001 (****). β-Actin was used as a loading control in the Western blot.

Overexpression and/or hyperactivation of AKT has previously been associated with cytoplasmic sequestration of CHK1, hence loss of its checkpoint activity that can ultimately lead to enhanced proliferation capacity with increased GI^24^. Since Cep55 overexpression increases Akt signaling, we investigated the impact of *Cep55* overexpression on replication by examining the replication fork progression rate using DNA fiber assay. We found that the *Cep55-*overexpressing MEFs exhibited a significant increase in replication fork speed (median speed: 1.47 kb/min) compared to wild-type cells (median speed: 1.03 kb/min) (Fig. 4a, b). On the contrary, transient silencing of *Cep55* in these cells significantly reduced replication fork speeds, suggesting that *Cep55* overexpression increases proliferation by allowing cells to replicate faster than the *Cep55*^*wt/wt*^ MEFs (Supplementary Fig. 6A, B). An increase in fork speed by 40% above the normal fork progression speed can induce DNA damage and genome instability^25^. Next, we investigated the impact of increased replication speed on DNA damage response in the *Cep55*^*Tg/Tg*^ MEFs. Interestingly, we initially observed that the *Cep55*^*Tg/Tg*^ MEFs exhibited a significantly higher percentage of γ-H2ax positive cells (>5 γ-H2ax foci per cell) when compared to the *Cep55*^*wt/wt*^ MEFs (Supplementary Fig. 6C, D). Likewise, we found that *Cep55*^*Tg/Tg*^ MEFs have a higher percentage of EdU-positive cells, compared to *Cep55*^*wt/wt*^ MEFs (Fig. 4c). Notably, an increase in the percentage of γ-H2ax positive cells was seen in both Edu-positive and Edu-negative population of the *Cep55*^*Tg/Tg*^ *MEFs*, suggesting that DNA damage is persistent (Fig. 4c). Despite this increase in baseline damage, no significant differences in DNA damage response signaling were apparent between these lines when these cells were challenged with 6-Gy γ-irradiation (Fig. 4d, Supplementary Fig. 6E). However, we noticed a marked reduction in total Chk1 levels in *Cep55*^*Tg/Tg*^ MEFs (Fig. 4d). ATR-dependent CHK1 is a well-established effector of DNA damage and replication stress response which is also required for faithful chromosome segregation^26^. Since *Cep55*^*Tg/Tg*^ MEFs have highly elevated Akt signaling (Fig. 3f), we initially investigated the subcellular Chk1 distribution in MEFs of different *Cep55* genotypes. Compared to *Cep55*^*wt/wt*^ MEFs, the *Cep55*^*Tg/Tg*^ MEFs show relatively higher Chk1 levels in cytoplasmic but reduced levels in nuclear fraction (Fig. 4e, left). Notably, treatment of *Cep55*^*Tg/Tg*^ MEFs either with Pi3k or Akt inhibitor markedly altered the localization of Chk1 from cytoplasmic to nuclear fraction, confirming that the activation of Akt signaling in *Cep55*-overexpressing cells sequesters Chk1 in the cytoplasmic fraction (Fig. 4e, right). To further confirm the involvement of an Akt-mediated replication stress, we treated *Cep55*^*Tg/Tg*^ MEFs with either BEZ235 or AKTVIII inhibitors and performed DNA fiber assay. Our data showed that treatment of *Cep55*-overexpressing cells with Akt inhibitors significantly reduced replication fork speeds compared to DMSO treated cells (Fig. 4f and g and Supplementary Fig. 6F). AKT phosphorylates CHK1 at serine 280 and impairs its nuclear localization and checkpoint activity independent of ATR^24^. To determine the crucial role of Cep55-Akt-dependent checkpoint deficiency, we transiently reconstituted *Cep55*^*Tg/Tg*^ cells with *S280A* mutant (that cannot be phosphorylated by active-AKT), and *Cep55*^*wt/wt*^ cells with *S280E* mutant (mimics constitutive AKT-dependent phosphorylation). Our data showed that while *S280E* mutant significantly increased replication fork speed in *Cep55*^*wt/wt*^ cells, the *S280A* mutant reconstituted *Cep55*^*Tg/Tg*^ cells on contrary show significantly decreased replication fork speed, suggesting that the checkpoint activity is impaired in Cep55-Akt-dependent manner in these cells (Fig. 4h, Supplementary Fig. 6G). Collectively, our data suggest that overexpression of *Cep55* impairs Chk1-mediated checkpoint activation leading to faster replicating cells with persistent DNA damage in our model.

### Cep55 overexpression promotes structural and numerical CIN

**Fig. 5:**
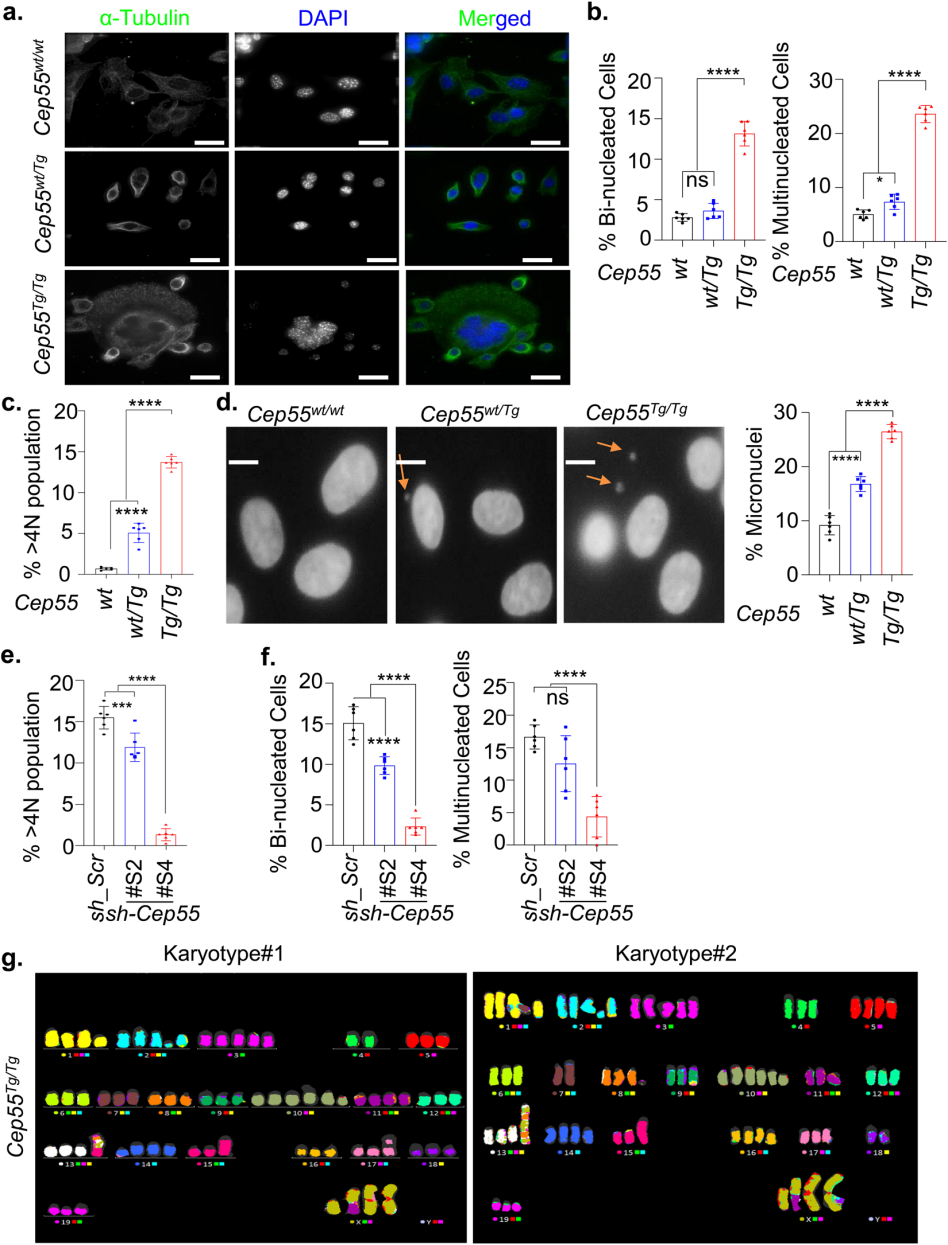
Cep55 overexpression promotes chromosomal instability in vivo. **a** Representative images of immunofluorescence demonstrating genomic instability observed in immortalized *Cep55*^*Tg/Tg*^ MEFs, as indicated by the presence of multiple nuclei (marked by DAPI staining) compared to other counterparts. The cell cytoplasm is marked by α-tubulin (green) (Scale bar, 100 μm). **b** Quantification of the percentage of binucleated (left panel) and multinucleated cells (right panel) observed in the indicated immortalized MEFs (*n* = 100 cells were counted of each genotype). Error bars represent the ± SD from three independent experiments with two replicates each. One-way ANOVA test was performed to determine *P-value;* < 0.0001 (****) and ns (not significant). **c** Quantification of percentage of polyploidy cells (>4 N DNA content) determined using FACS analysis in the indicated immortalized MEFs. Error bars represent the ± SD from three independent experiments with two replicates each. One-way ANOVA test was performed to determine *P-value;* < 0.0001 (****). **d** Representative images showing the presence of micronuclei (marked by DAPI) in the indicated immortalized MEFs (left panel) (Scale bar, 100 μm). The micronuclei were counted manually per field of view/per nuclei, *n* = 300 cell were counted per experiment and the percentage of micronuclei observed in the indicated immortalized MEFs is shown in (right panel). Error bars represent the ± SD from three independent experiments with two replicates each. One-way ANOVA test was performed to determine *P-value;* < 0.0001 (****). **e** Percentage of polyploidy population (>4N DNA content) determined using FACS in the respective *shCep55* depleted isogenic clones. Error bars represent the ± SD from three independent experiments with two replicates each. One-way ANOVA test was performed to determine *P-value;* < 0.001 (***) and < 0.0001 (****). **f** Quantification of the percentage of binucleated (left panel) and multinucleated cells (right panel) observed in the respective sh*Cep55* depleted isogenic clones. Error bars represent the ± SD from three independent experiments with two replicates each (*n* = 100 cells per clone were counted). One-way ANOVA test was performed to determine *P-value;* < 0.0001 (****) and ns (not significant). **g** Representative metaphases from spectral karyotyping (SKY) in the *Cep55*^*Tg/Tg*^ MEFs (passage 25) wherein #1 and #2 denotes biologically independent metaphase representation of immortalized *Cep55*^*Tg/Tg*^ MEFs.

The well-known role of CEP55 as a regulator of CIN is through the regulation of cytokinesis^3^. Consistent with this, we found that whole-genome duplicated (WGD) tumors have significantly higher levels of *CEP55* mRNA than diploid and near-diploid tumors (Supplementary Fig. 7A). Likewise, immortalized *Cep55*^*Tg/Tg*^ MEFs exhibited a three-fold higher percentage (*p* < 0.0001) of binucleated and multinucleated cells (Fig. 5a, b). In addition, using FACS analysis, we found that both primary and immortalized *Cep55*^*Tg/Tg*^ MEFs exhibited a significantly higher percentage of >4*n* subpopulation (Fig. 5c, Supplementary Fig. 7B). We also observed that compared to the primary *Cep55*^*Tg/Tg*^ MEFs, the spontaneously immortalized *Cep55*^*Tg/Tg*^ MEFs comprised significantly higher percentage (*p* < 0.01) of >4*n* subpopulation (Supplementary Fig. 7C). Similar results were observed in different organs isolated from *Cep55*^*Tg/Tg*^ mice compared to their littermate counterparts (Supplementary Fig. 7D). Importantly, we found a significant increase in micronuclei in the *Cep55*^*Tg/Tg*^ MEFs (*p* < 0.001) indicating the possible presence of CIN (Fig. 5d). Likewise, when *Cep55* was constitutively knocked down in TCLs, we found a significant reduction in >4*n* subpopulations (Fig. 5e, f, Supplementary Fig. 7E), suggesting that Cep55 overexpression contributes to cancer cells ability to tolerate aneuploidy as reported previously by us for breast cancer cells^18^. Consistent with this, when we analyzed the level of aneuploidy across some of the human cancers using Genome-wide SNP6 array data from TCGA, we found that *CEP55* overexpressing tumors show increased structural or numerical aneuploidy, including whole-chromosome aneuploidy and chromosome arm-level aneuploidy (Supplementary Fig. 8A–D). Additionally, spectral karyotyping of metaphase spreads from *Cep55*^*Tg/Tg*^ MEFs demonstrated the presence of significantly higher levels of both numerical and structural chromosomal aberrations compared to other genotypes (Fig. 5g). Notably, these MEFs demonstrated complex chromosomal translocations and numerical abnormalities, whereas both *Cep55*^*wt/Tg*^ and *Cep55*^*wt/wt*^ MEFs showed a low level of structural and numerical chromosomal abnormalities (Table 2). In summary, these data highlight that Cep55 overexpression above a certain threshold is sufficient to promote structural and numerical CIN.

**Table 2:** Changes in chromosomal alterations in *Cep55* transgenic MEFs.

Genotype	Karyotype	Phenotype
*Cep55*^*wt/wt*^	77,XXXX,-6,-7,-18[17]	Hypotetraploid with numerical abnormalities.
*Cep55*^*wt/Tg*^	80,XXXX[6]/77,idem,-6,-7,-18[11]/40,XX[4]	Four normal female metaphases. Six tetraploid metaphases and eleven hypotetraploid metaphases with the same numerical abnormalities that were seen in the WT cell line.
*Cep55*^*Tg/Tg*^	72~74,X,der(X)t(X;11)(F?1;A?2),i(X)(A1)x2,del(1)(A?E?),del(2)(?B?H), + 3,-4,-6,-7,del(8)(A?2),-9,der(9)(9pter->9?F::2??2?F::1?H > 1qter)[3],der(9)t(9;17)(F?;E?1)[2],+10,+10,del(10)(A2B4)x3,-11,-12,der(13)(13pter->13?::8?->8?::13?->13?:: 8?->8?:: 13?->13qter)[12],der(13) (13pter->13?::8?->8?::13?->13?:: 8?->8?:: 13?->13?::5?->5qter)[2],	Hypotetraploid with complex numerical and structural abnormalities.
	der(13) (13pter->13?::8?->8?::13?->13?:: 8?->8?:: 13?->13?::15?->	
	5qter)[3],der(13)t(13;14)(A?;B?)[2],-15,dup(15)(ED?2),-17,der(17)	
	t(9;17)(?F1;?B)[3],i(17)(A1),-18,-19[cp17]	

? = questionable identification of chromosome or chromosome structure.

### Cep55 overexpression delays mitotic exit

**Fig. 6:**
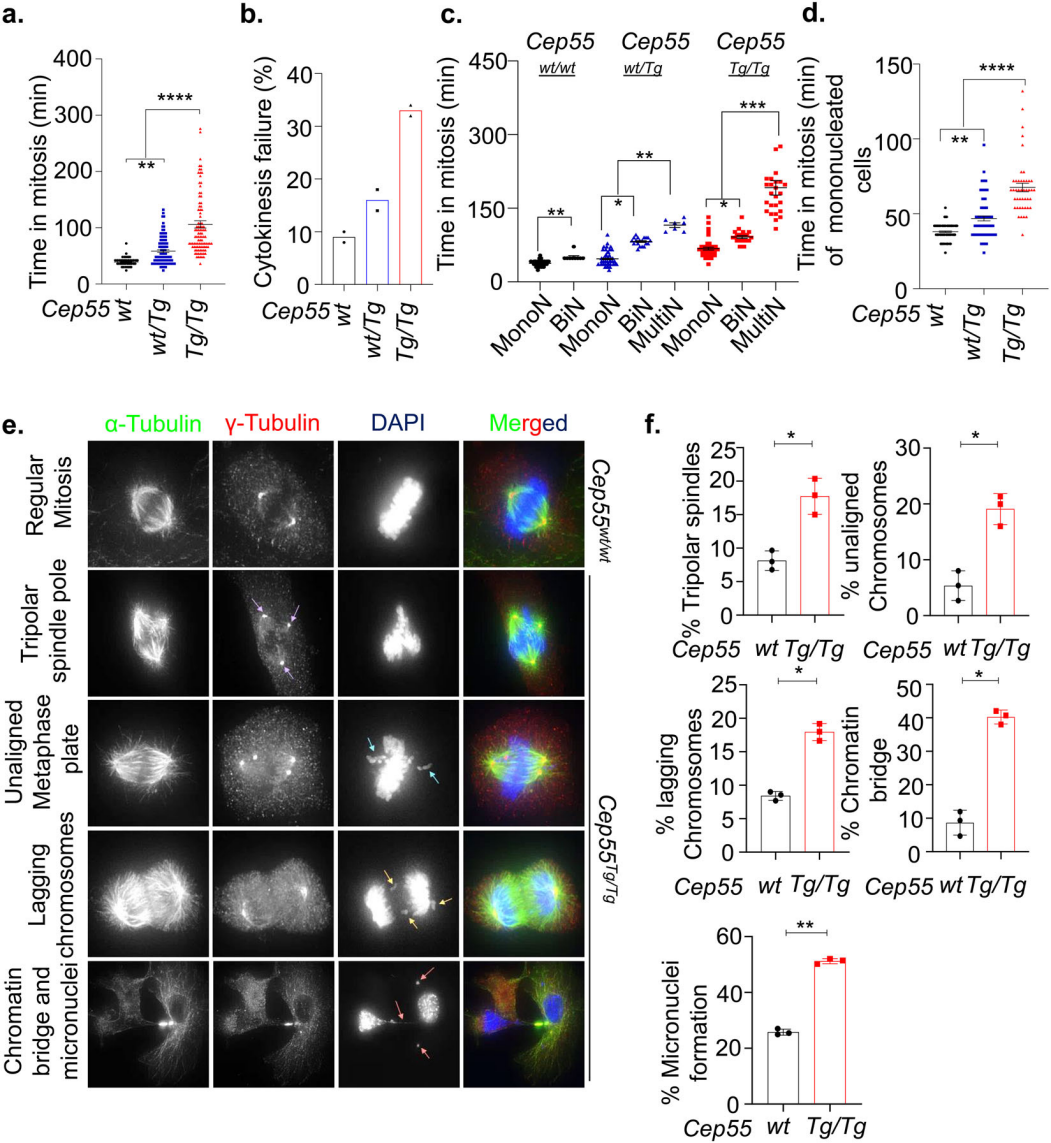
Impact of Cep55 overexpression on mitosis. **a** Quantification of the average time spent in mitosis by the immortalized MEFs of indicated genotypes. MEFs were synchronized using double‐thymidine and released into regular media. Individual cells were tracked using bright-field Olympus Xcellence IX81 time-lapse microscopy for overall time taken to complete mitosis from nuclear envelope breakdown up to daughter cell formation^18^. At least 50 cells were counted per experiment. Error bars represent the ± SD from two independent experiments with three replicates each. One-way ANOVA test was performed to determine *P-value;* < 0.01 (**) and < 0.0001 (****). **b** Boxplots showing the percentage of cytokinesis failure observed in the immortalized MEFs of indicated genotypes. Data derived from experiment (**a**). Percentage was derived from 50 cells per experiment. **c** Quantification of average time spent in mitosis by different cell populations observed among the immortalized MEFs of indicated genotypes. At least 100 cells were counted. Error bars represent the ± SD from two independent experiments with three replicates each. Two-way ANOVA test was performed to determine *P-value* < 0.05 (*), < 0.01 (**) and < 0.001 (***). **d** Quantification of average time spent in mitosis by immortalized mononucleated MEFs of indicated genotypes. Error bars represent the ± SD from two independent experiments as described above. One-way ANOVA test was performed to determine *P-value* < 0.01 (**) and < 0.0001 (****). **e** Representative images of immunofluorescence (Scale bar, 100 μm) and (**f**) statistical analyses of the mitotic defects observed in the immortalized *Cep55*^*wt/wt*^ and *Cep55*^*Tg/Tg*^ MEFs as indicated by the presence of tripolar spindle poles, unaligned metaphase plates, lagging chromosomes, as well as chromatin bridges and micronuclei. The arrows represent the different mitotic phenotype observed across immortalized *Cep55*^*Tg/Tg*^ MEFs. The blue arrow represents unaligned chromosomes, the yellow arrows represent presence of lagging chromosomes, the purple arrows represent tripolar spindle and orange arrows represent the presence of chromatin bridge and micronuclei. Error bars represent the ± SD from two independent experiments with three replicates each. *n* = 50 mitotic cells were counted per experiment. Student’s *t* test was performed to determine *P-value*; < 0.05 (*) and < 0.01 (**).

CIN in cancers primarily occurs due to defective mitosis including unequal chromosome segregation and failure to undergo cytokinesis. Our initial analysis of percentage of cells undergoing mitosis revealed that *Cep55*^*Tg/Tg*^ MEFs had a significantly increased mitotic index compared to other genotypes (Supplementary Fig. 9A, B; *p* < 0.001) and *Cep55*-depleted TCLs showed a reduction in the number of mitotic cells (Supplementary Fig. 9C). We next asked how *Cep55* overexpression might promote both structural and numerical CIN in these cells during normal and perturbed mitosis. To decipher this, we collected double-thymidine synchronized MEFs for DNA content and time-lapse live-cell imaging analyses. Notably, we observed that the *Cep55*^*Tg/Tg*^ MEFs progressed faster through interphase and entered mitosis more rapidly compared to *Cep55*^*wt/wt*^ MEFs (Supplementary Fig. 9D). However, the *Cep55*^*Tg/Tg*^ MEFs spent a relatively longer time in mitosis with a higher percentage of cells exhibiting cytokinesis failure compared to wild-type and heterozygous MEFs (Fig. 6a, b). Likewise, the *Cep55*^*wt/Tg*^ MEFs also spent significantly more time in mitosis compared to wild-type MEFs, indicating a dosage-dependent impact of *Cep55* overexpression on mitotic duration (Fig. 6a). Multinucleated cells usually take more time to complete mitosis due to high DNA content and the *Cep55*^*wt/Tg*^ and *Cep55*^*Tg/Tg*^ MEFs exhibited mixed subpopulations of mononucleated, binucleated, and multinucleated cells (Fig. 5b, c and Supplementary Fig. 9E). We therefore performed analysis of individual subpopulations to determine the duration of mitosis (Fig. 6c). Surprisingly, along with the bi- and multinucleated *Cep55*^*Tg/Tg*^ cells, the mononucleated cells also spent more time in mitosis, indicating that *Cep55* overexpression prolonged mitotic duration independently of DNA content (Fig. 6d). Chromosome segregation errors are a major source for CIN^27^. Next, we investigated the impact of *Cep55* overexpression on chromosome segregation using cells synchronized in mitosis. We observed a significantly higher frequency (*p* < 0.05) of multipolar spindle poles along with unaligned and lagging chromosomes in *Cep55*^*Tg/Tg*^ MEFs compared to *Cep55*^*wt/wt*^ MEFs (Fig. 6e, f, Supplementary Fig. 10A, B). In addition, using both fluorescence and live-cell time-lapse microscopy, we also observed that the *Cep55*^*Tg/Tg*^ MEFs showed a significantly higher frequency of anaphase cells with chromatin bridges (anaphase bridges). The presence of anaphase bridges during mitosis indicates the presence of incompletely segregated DNA in *Cep55*^*Tg/Tg*^ MEFs which in turn result in chromosomal breakage and micronuclei formation (Fig. 6f). Consistent with this, we observed that the *Cep55*^*Tg/Tg*^ MEFs exhibited an increased proportion (*p* < 0.001) of micronuclei, a morphological characteristic of CIN, when compared to control MEFs (Fig. 6f).

### Cep55 overexpression stabilizes microtubules

**Fig. 7:**
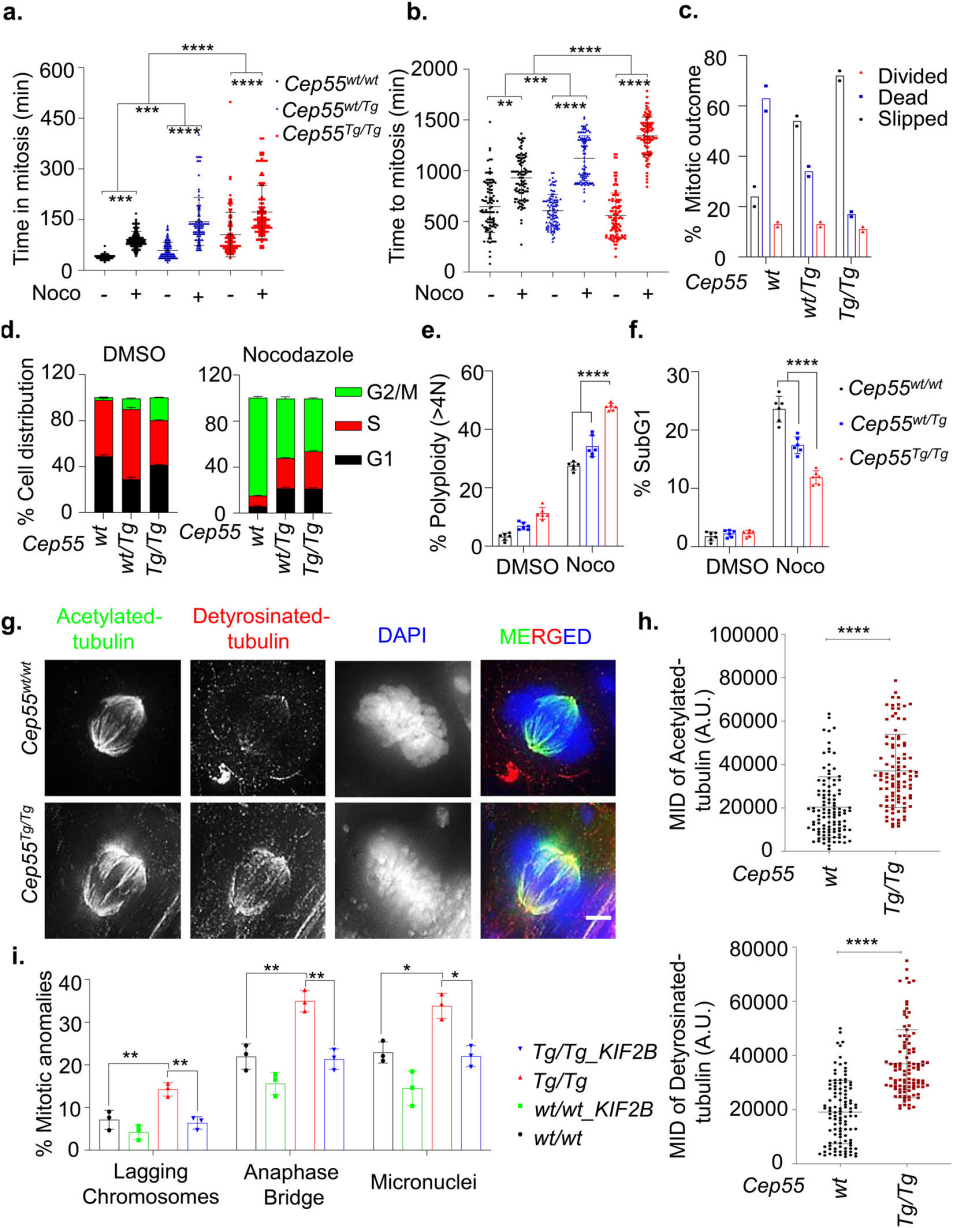
Association of CEP55 with microtubule stability. **a**, **b** Comparison of average time spent in mitosis (**a**) and average time taken to complete mitosis (**b**) determined using time-lapse microscopy of the immortalized MEFs of indicated genotypes in the presence and absence of nocodazole (0.5 μM). Error bars represent the ± SD from two independent experiments (fate of *n* = 50 cell were counted per experiment). Student’s *t* test was performed to determine *P*-value < 0.05 (*), < 0.01 (**), < 0.001 (***) and < 0.0001 (****). **c** Percentage of mitotic outcome of the immortalized MEFs of indicated genotypes in presence of nocodazole (0.5 μM) as shown in panel A and B. Mitotic slippage was defined by premature mitotic exit during nocodazole-induced mitotic arrest, while death was determined through membrane blebbing. Mean derived from two independent experiments (fate of *n* = 50 cell were counted per experiment). **d** Cell-cycle profiles of immortalized MEFs of indicated genotype in the presence or absence of nocodazole (0.5 μM) determined using FACS. Error bars represent the ± SD from three independent experiments. Two-way ANOVA test was performed to determine P-value as demonstrated in Supplementary Table 3. **e**, **f** Polyploidy (>4N DNA content) (**e**) and percentage of SubG1 populations (**f**) determined using FACS in the indicated immortalized MEFs in the presence or absence of nocodazole (0.5 μM). Error bars represent the ± SD from three independent experiments with two replicates each. One-way ANOVA test was performed to determine *P*-value < 0.0001 (****). **g** Representative images of detryosinated (red) and acetylated tubulin (green) of metaphase stages of immortalized *Cep55*^*wt/wt*^ and *Cep55*^*Tg/Tg*^ MEFs showing the microtubule networks (Scale bar, 100 μm). **h** Quantification of the mean integrated density (MID) of Acetylated (Upper panel) and detyrosinated (lower panel) tubulin observed among immortalized *Cep55*^*wt/wt*^ and *Cep55*^*Tg/Tg*^ MEFs. The intensity was calculated using Image J software wherein *n* = 50 metaphase cells were calculated per genotype. Error bars represent the ± SD from two independent experiments. Student’s *t* test was performed to determine *P*-value < 0.0001 (****). **i** Percentage of mitotic defects (described previously in Fig. 6f upon KIF2B overexpression in the immortalized MEFs (*n* = 100 cell per experiment were counted) of indicated genotypes. Error bars represent the ± SD from three independent experiments. One-way ANOVA test was performed to determine *P*-value < 0.05 (*) and < 0.01 (**).

We recently reported that CEP55 overexpression causes premature exit during perturbed mitosis and is determinant of aneuploid breast cancer cell survival^18^. Consistent with our previous observation, Cep55 overexpression significantly impacted the duration of time to- and time spent in mitosis upon nocodazole treatment (Fig. 7a, b). In particular, the *Cep55*^*Tg/Tg*^ MEFs largely prematurely exited mitosis during nocodazole arrest but the *Cep55*^*wt/wt*^ MEFs predominately died in mitosis, despite an increase in expression of components of spindle assembly checkpoint (SAC) proteins, Bubr1 and Mad2 levels in *Cep55*^*Tg/Tg*^ compared to *Cep55*^*wt/wt*^ MEFs (Fig. 7c–f, Supplementary Fig. 11A). Additionally, we found that SAC activity was not impaired after nocodazole treatment (4 h), as assessed by assembly of SAC protein complex (Cdc20. Bubr1 and Mad2) (Supplementary Fig. 11B). In contrast, the *Cep55*-depleted TCL showed sensitivity towards nocodazole treatment with a significant reduction in premature exit and increase in apoptosis (Supplementary Fig. 11C–E). Therefore, these data indicate that *Cep55* overexpression facilitates mitotic slippage rather than death in response to anti-mitotic poisons irrespective of normal activation of SAC. This is consistent with our previous study wherein we showed that CEP55 overexpression confers resistance to anti-mitotic poisons despite prolonged activation of SAC through the inability of cells to breach apoptotic threshold^18^.

Increased kinetochore-microtubule (k-MT) stability causes incomplete segregation of DNA, including lagging chromosomes during anaphase^28,29^. As CEP55 is recruited to spindle microtubules during mitosis^3^ and efficiently bundles microtubules^30^, we asked if *Cep55* overexpression stabilizes microtubules, and hence increasing segregation errors during mitosis. To analyze spindle microtubule stability, mitotic cells were stained with antibodies that recognize stable detyrosinated- and acetylated-microtubules. *Cep55*^*Tg/Tg*^ mitotic cells exhibited enhanced detyrosinated- and acetylated- microtubule staining compared to mitotic *Cep55*^*wt/wt*^ cells, indicating these cells have stabilized microtubules in metaphase or midbodies (Fig. 7g, h, Supplementary Fig. 11F). Next, to confirm that increased chromosome segregation errors, including lagging chromosomes, in response to *Cep55* overexpression are due to stabilized microtubules, we expressed GFP-tagged *KIF2B*, microtubule depolymerizing kinesin-13 protein, in both *Cep55*^*Tg/Tg*^ and *Cep55*^*wt/wt*^ MEFs. In particular, exogenous expression of *KIF2B* in *Cep55*^*Tg/Tg*^ cells significantly reduced the frequencies of lagging chromosomes, anaphase bridges, and micronuclei (Fig. 7i), Likewise, treatment of these cells with low concentration of nocodazole at 10 nM significantly reduced percentage of lagging and unaligned chromosomes with increase in normal mitosis (Supplementary Fig. 11F, G). Collectively, our data suggest that overexpression of *Cep55* stabilizes microtubules that in part lead to the mitotic defects observed in these MEFs.

## Discussion

We have previously reported a *Cep55*-overexpression mouse model that exhibits male-specific sterility by suppressing Foxo1 nuclear retention through hyperactivation of Pi3k/Akt signaling^17^. In this study, using the same mouse model, to the best of our knowledge we demonstrate for the first time that *Cep55* overexpression causes spontaneous tumorigenesis. Our data highlight the dosage-dependent impact of *Cep55* overexpression on cell proliferation and tumorigenesis in vivo. The homozygous *Cep55*^*Tg/Tg*^ mice are prone to develop a wide spectrum of tumors (both solid and hematological origin) with a high incidence rate and high metastatic potential. Interestingly, heterozygous *Cep55*^*wt/Tg*^ mice developed a lower percentage of adenomas (~20%) and hyperplasia (~8%), suggesting that single copy overexpression of *Cep55* is sufficient to initiate tumorigenesis, although the latency significantly differs between *Cep55*^*Tg/Tg*^ and *Cep55*^*wt/Tg*^ mice. Notably, the *Cep55*^*Tg/Tg*^ mice demonstrated a higher incidence of lymphomas and sarcomas compared to other types of malignancies, mimicking the phenotype observed in *Trp53*^*−/−*^ mice. As p53 negatively controls CEP55 expression^13^, using a bi-transgenic mouse model, we also demonstrated that single copy loss of p53 (*Trp53*^*+/−*^*)* accelerates heterozygous *Cep55*^*wt/Tg*^ *-*induced tumorigenesis. Interestingly, these data also illustrate that either loss or mutation of *Trp53* might be an early event and a critical secondary hit is required for tumor initiation observed in the homozygous *Cep55*^*Tg/Tg*^ mice. Consistent with this, we observed high p53 protein levels, which are most likely an indication of mutated *Trp53*, in representative *Cep55*^*Tg/Tg*^ tumor tissues than normal adjacent tissues. Notably, partial depletion of *Cep55* (50%) in TCLs significantly delayed tumor initiation and progression, while near-complete depletion (90%) totally impaired tumor initiation in a xenograft model.

As *Cep55* has been linked with GI and its overexpression causes a wide range of tumors in vivo, we further characterized GI in *Cep55*-overexpressing cells. *Cep55*^*Tg/Tg*^ MEFs exhibited a high level of cytokinesis failure accompanied by genome doubling. Importantly the *Cep55*^*Tg/Tg*^ MEFs showed high level of numerical and structural CIN compared to MEFs of other genotypes. Importantly, in this study, we showed that *Cep55* overexpression causes mitotic defects including a high frequency of chromatin bridge and micronuclei formation during anaphase. As CEP55 is a microtubule-bundling protein^30^, missegregation of chromosomes upon *Cep55* overexpression might be indicative of kinetochore-microtubule (k-MT) hyper-stability. Consistent with this notion, we found that overexpression of *Cep55* stabilized microtubules and predisposed cells to CIN. Notably, reducing microtubule stability by forced expression of KIF2B in *Cep55*^*Tg/Tg*^ MEFs significantly reduced lagging chromosomes. The influence of *Cep55* overexpression on sister chromatid segregation errors accompanied by cytokinesis failure explains the delayed mitotic exit observed in the *Cep55*-overexpressing cells. Taken together, our data suggest that hyperstabilised microtubules and defective cytokinesis in Cep55-overexpressing cells might be major source of chromosome segregation errors and tetraploidization that can predispose these cells to GI which over time might facilitate tumor development.

Consistent with previous reports (reviewed by Jeffery et al.^4^.), *Cep55* overexpression led to rapid proliferation. We observed that the *Cep55*^*Tg/Tg*^ MEFs displayed hyper-phosphorylated Akt and deregulated downstream Pi3k/ Akt signaling such as Gsk-3β, Myc, and β-Catenin which might be a further source of GI in these cells. Akt hyperactivation is known to result in cytoplasmic sequestration of Chk1, this might result in a compromised S-phase checkpoint that increases replication fork progression in *Cep55*^*Tg/Tg*^ MEFs to allow uncontrolled cell cycle progression and consequently promote GI. Consistent with this, overexpression of CHK1 mutant (S280A), that cannot be phosphorylated by overactive AKT, in *Cep55*^*Tg/Tg*^ MEFs or their treatment with Pi3k/Akt pathway inhibitors resulted in reduced fork progression. Furthermore, loss of Chk1 function has also been shown to induce chromosomal segregation errors and chromatin bridges during anaphase resulting in CIN^26,31^, resembling the phenotype we observe.

Deregulation of mitotic proteins has long been known to contribute to early cellular transformation and tumorigenesis^32^ though they are rarely mutated in cancer^33,34^, but rather prone to amplification. Abnormal expression (loss or gain) of critical mitotic proteins, especially those included in the CIN70 gene signature, such as *MAD2*^35^, *BUB1*^36^, *AURKA*^37^, *EMI1*^38^, *PLK1*^39,40^, *TTK1*^41^ and many more, at the genetic level have been shown to induce spontaneous tumorigenesis. The major phenotype observed in these mouse models was defective chromosomal segregation during anaphase which led to CIN and genomically unstable malignancies, similar to the phenotype observed in our model. Thus, the interplay of these mitotic genes with *Cep55* overexpression needs further evaluation. Importantly, in our previous study in breast cancer, we have shown that CEP55 overexpression protects aneuploid cells during perturbed mitosis^17^. We have demonstrated that high level of CEP55 significantly induced mitotic slippage in TNBCs as loss of CEP55 enables mitotic cell death by enabling premature mitotic entry upon being challenged with anti-mitotic drugs. Consistently, herein we have demonstrated that Cep55 is a protector of aneuploidy during aberrant mitosis as the aneuploid *Cep55*^*Tg/Tg*^ MEFs underwent mitotic slippage in response to anti-mitotic drugs and survived mitotic cell death. It also explains the ability of the highly polyploid *Cep55*^*Tg/Tg*^ MEFs to re-enter mitosis and continue proliferation as CEP55 overexpression allows high tolerance and better survival of these cell populations.

A recent report has suggested that cells procure specific genomic alterations, mainly impacting the regular function of mitotic genes prior to malignant transformation^42^. *CEP55* overexpression has been linked with tumorigenesis for a wide variety of cancers. However, this is the first report to our knowledge demonstrating that overexpression of *Cep55* has a causative role in tumorigenesis. Our data clearly demonstrate that *Cep55* overexpression beyond a critical level is self-sufficient to induce a wide spectrum of spontaneous tumors. Importantly, we have shown that *Cep55* overexpression leads to the induction of pleotropic events such as Pi3k/Akt pathway activation, Chk1 sequestration compromising the replication checkpoint, and stabilized microtubules along with chromosomal segregation anomalies which all together cause CIN. Accumulation of these anomalies over time might induce tumourigenesis. In summary, our mouse model could be a valuable tool in studying the mechanism of CIN-associated tumorigenesis and development of CIN-targeting therapies.

## Methods

### Reagents

Nocodozole, BEZ235, BKM120, AZD6244 and AKTViii were purchased from Selleck Chemicals LCC. Small interfering RNAs (siRNAs) were from Shanghai Gene Pharma. Dulbecco’s Modified Eagle’s Media (DMEM), Click-iT Alexa Fluor 488 EdU (5-ethynyl-2’-deoxyuridine) imaging kit and Lipofectamine RNAiMAX was purchased from Life Technologies. Foetal Bovine Serum (FBS) was purchased from SAFC Biosciences™, Lenexa, USA. CellTiter 96^®^ AQueous One Solution Cell Proliferation Assay and Dual-Glo® Luciferase Assay were purchased from Promega Corporation.

### Animal husbandry and ethics statement

All animal work was approved by the QIMR Berghofer Medical Research Institute, Animal Ethics Committee (number A0707-606M) and was performed in strict accordance with the Australian code for the care and use of animals for scientific purposes. The animals were maintained as per the guidelines^17^.

### Histopathological analysis and immunohistochemistry

For histologic examination, tissues were collected and fixed in 4% formaldehyde in PBS as per the standard protocol described by Sinha et al.^17^. Briefly, the tissues were embedded in paraffin blocks, and 5-μm-thick sections prepared for respective stains after being fixed in 4% formaldehyde. Immunostaining was performed with the following primary antibodies: Ki67 1:500 (anti-rabbit, Novacastra #NCL-ki67p), B220 1:500 (anti-rat, ThermoFischer Scientific #14-0452-82), CD3 1:250 (anti-rabbit, Abcam #ab5690), p21 1:500 (anti-rabbit, Abcam #ab188224), p53 1:400 (anti-rabbit, Abcam #ab131442).

### Cell culture and synchronization

To generate the MEFs, mice pregnancy was accessed on the basis of a copulation plug on the following morning post-mating date, designated as embryonic day. Such assessment was done for isolating MEFs on E13.5 and single-cell isolation was performed using the standard protocol^43^. Continuous passaging every 48 h in culture immortalized the MEFs of each genotype. To generate the primary tumor lines (TCLs), tumor was surgically removed followed by mechanical disaggregation using a sterile scalpel blade and then incubation in 0.1% collagenase (Sigma Aldrich) in 10 mL of DMEM containing 20% FBS and 1% penicillin-streptomycin (100 U/mL), 1% L-glutamine and cultured in a 25 cm^2^ tissue flasks. After 24 h, the cells were trypsinized and cultured in a new 25 cm^2^ tissue flask with media supplemented with 100 µL (100 µg/mL) of EGF, 500 µL (10 mg/mL) of insulin and 1% Sodium pyruvate (Life Technology^TM^. The culture of the murine cell lines was maintained by incubating at 37 °C with 20% oxygen levels and 5% CO_2_. Cells were synchronized at G1/S by double-thymidine block^44^.

### Genotype analysis and quantitative real-time PCR

Genotyping, RNA extraction, and quantitative real-time PCR was performed using the primer sets used in these assays were used as per standard protocol^17^. Briefly, total RNA from respective cells of each genotype was isolated using an RNeasy plus mini kit (Qiagen). 2 μg of RNA was used for first-stand cDNA synthesis using random primers (Life Technologies) and Superscript III reverse transcriptase (Life Technologies). qRT-PCR was performed using Light Cycler 480 Sybr green mastermix (Roche Applied Science) on a Light Cycler 480 real-time PCR cycler (Roche Applied Science).

### Immunoblotting and Immunoprecipitation

The protein extraction from cell lysate or tissue lysate was prepared in urea lysis buffer (8 M urea, 1% SDS, 100 mM NaCl, 10 mM Tris (pH 7.5) and incubated for 30 min on ice after which the samples were sonicated for 10 seconds. Western blotting was performed as per the standard protocol and some of the antibodies used for immunoblotting^17^. The following are additional antibodies used in this study: Cell Signaling antibodies: PARP (#9542), pAKT^S473^ (#4060), AKT (#9272), pPdk1^S241^ (#3061), Pdk1(#3062) Chk1 (2G1D5) (#2360), p-GSK-3β^(Ser9)^ (#9336), GSK-3β (#9315), p-Histone H3 (#9706) (1:1000 dilution); Millipore antibody: Chk2 (1:500 dilution) (Clone 7) (05-649), γ-H2ax (1:1000 dilution) S139 (05-636); BD Pharmingen antibody: β-actin (1:2000 dilution) (612656); Bethyl antibody: pKap1^(S824)^ (1:1000 dilution) (A300-767A). Immunoprecipitation was performed as per our previous publication^45^. Immunodetections were performed using Bubr1 (ab4637), Mad2 (CST4636S) and CDC20 (CST14866A).

### Cell proliferation assay

The cell proliferation assay using The IncuCyte® S3 Live-Cell Analysis system (Essen BioSciences Inc, USA), as described by Kalimutho et al.^18^. Doubling time was analyzed at every 12 h interval by counting the overall cell population compared to the originally seeded population using the Countess^®^ automated cell counter (Life Technologies^TM^). The NIH-3T3 proliferation assay was performed by using the standard protocol^43^.

### Colony formation assays

Five hundred to one thousand cells were seeded on 12 well plates and incubated for additional 14 days to determine colony viability. The colonies were fixed with 0.05% crystal violet for 30 min^18^.

### Flow cytometry and cell cycle analysis

Cell-cycle perturbations and the subG1 apoptotic fractions were determined using flow cytometry analysis of cells stained with propidium iodide and analyzed using the ModFit LT 4.0 software^18^.

### Immunofluorescence

Cells were seeded and incubated overnight on coverslips and were fixed for 15 min in 4% paraformaldehyde in PBS, permeabilized in 0.5% Triton X-100-PBS for 15 min and blocked in 3% filtered bovine serum albumin (BSA) in PBS. Coverslips with primary antibodies were diluted in blocking solution and incubated overnight at 4 °C. Alexafluor conjugated secondary antibodies were diluted 1:300 and DAPI (diluted 1:500 in blocking buffer, stock 1 mg/ml), in blocking solution and stained for 45 min at 37 ^o^C in humidifier chamber. Slides were washed thrice with 0.05% Tween 20 in PBS and mounted in Prolong Gold. Slides were imaged using GE DeltaVision Deconvolution microscope and analyzed using Image J. Antibodies used for immunofluorescence were: γ-H2ax S139 (05-636; Millipore), p-Histone H3 (#9706; CST), α-Tubulin (T9026), γ-Tubulin (T5192), Acetylated tubulin (T7451; Sigma) and detyrosinated tubulin (ab48389; Abcam).

### DNA combing assay

The DNA fiber protocol was followed as described previously by us and others^46,47^. Briefly, cells were pulse-labeled with CldU and IdU for 20 min each. Progressive replication fork speed was calculated based on the length of the IdU (red) tracks measured using ImageJ software. At least 300 replication tracks were analyzed for each sample in two independent experiments. The fork speed was calculated based on conversion factor 1 µm = 2.59 kb^48^.

### Gene silencing

Transient gene silencing was performed by reverse transfection using 10 nM of respective small interfering RNAs (siRNAs). The sequences involved *Cep55*_Scr (5′CAAUGUUGAUUUGGUGUCUGCA3′); *Cep55*_SEQ1 (5′ CCAUCACAGAGCAGCCAUUCCCACT 3′) and *Cep55*_SEQ2-targeting *UTR* (5′ AGCUACUGAGCAGUAAGCAAACAUU). The siRNAs were manufactured by Shanghai Gene Pharma. The transfection was performed using Lipofectamine RNAiMAX (Life Technologies^TM^). Mouse small hairpin RNAs (shRNAs) for Cep55 (pLKO plasmids, (Sigma Aldrich^®^, St Louis, USA)) clones were established using lentiviral packaging using PEI (Poly -ethyleneimine) solution (Sigma Aldrich^®^, St Louis, USA).

### Cep55_Scr

(5′CCGGCGCTGTTCTAATGACTAGCATCTCGAGATGCTAGTCATTAGAACAGCGTTTTTT′3);

### Cep55_sh#1: TRCN0000366894 CDS

(5′CCGGCCGTGACTCAGTTGCGTTTAGCTCGAGCTAAACGCAACTGAGTCACGGTTTTTG);

### Cep55_sh#2 TRCN0000366948 CDS

(5′CCGGCAGCGAGAGGCCTACGTTAAACTCGAGTTTAACGTAGGCCTCTCGCTGTTTTTG3′);

### Cep55_sh#3 TRCN0000183083 CDS

(5′CCGGCGTTTAGAACTCGATGAATTTCTCGAGAAATTCATCGAGTTCTAAACGTTTTTTG3′);

### Cep55_sh#4 TRCN0000183560 CDS

(5′CCGGGAAGATTGAATCAGAAGGTTACTCGAGTAACCTTCTGATTCAATCTTCTTTTTTG3′).

### Live cell imaging

Live cell imaging for double thymidine releases was performed on an Olympus IX81 microscope using excellence rt v2.0 software. Images were analyzed using analySIS LS Research, version 2.2 (Applied Precision)^49^. Live cell imaging for tracking mitotic defects was performed in H2B Cherry transfected MEFs of each genotype using 20X Andor Revolution WD - Spinning Disk microscope.

### In vivo xenografts

All mice were housed in standard condition with a 12 h light/dark cycle and free access to food and water. 2.5 × 10^6^ TLC were prepared in 50% matrigel (BD, Biosciences, Bedford, USA)/PBS and injected subcutaneously injected into the right flank of 6-week-old NOD/SCID mice^18^.

### Bioinformatics analysis

Whole-chromosome (WC) and chromosome arm-level (CAL) somatic copy number aberrations (SCNAs) were inferred from TCGA processed (Level 3) Affymetrix Genome Wide SNP6.0 Array data for the indicated cancer types, as previously described^50^. Using the same datasets, ASCAT2.4^51^ was used to compute the ploidy level for each sample. Samples with ploidy between 1.9 and 2.1 were considered diploid, samples with ploidy lower than 1.9 or between 2.1 and 2.5 were called near-diploid aneuploid and samples with ploidy>2.5 were considered aneuploid and having undergone at least one whole-genome doubling (WGD)^52,53^.

### Statistics and reproducibility

Student’s *t* test; one-way or two-way ANOVA; RPKM and RSEM with Bonferoni *post hoc* or Mann-Whitney *U* test testing (specified in figure legend) and Fisher exact test was performed using GRAPHPAD PRISM v6.0 (GraphPAd Software, LaJolla, CA, USA) and the p-values were calculated as indicated in figure legends. Asterisks indicate significant difference (*p < 0.05, **p < 0.01, ***p < 0.001 and ****p < 0.0001), ns = not significant.

### Reporting summary

Further information on research design is available in the Nature Research Reporting Summary linked to this article.

## Supplementary information

Supplementary Information

Description of Additional Supplementary Files

Supplementary Data 1

Reporting Summary

## Data Availability

The datasets generated and/or analyzed during the current study are included in this published article (and its supplementary information files) and all the raw data available from the corresponding author on reasonable request. Source data can be found in Supplementary Data 1.
