# Supplementary material for: Cep55 overexpression promotes genomic instability and tumorigenesis in mice
Source: Commun Biol. 2020 Oct 21;3:593. doi: 10.1038/s42003-020-01304-6 (PMC7578791; doi:10.1038/s42003-020-01304-6)

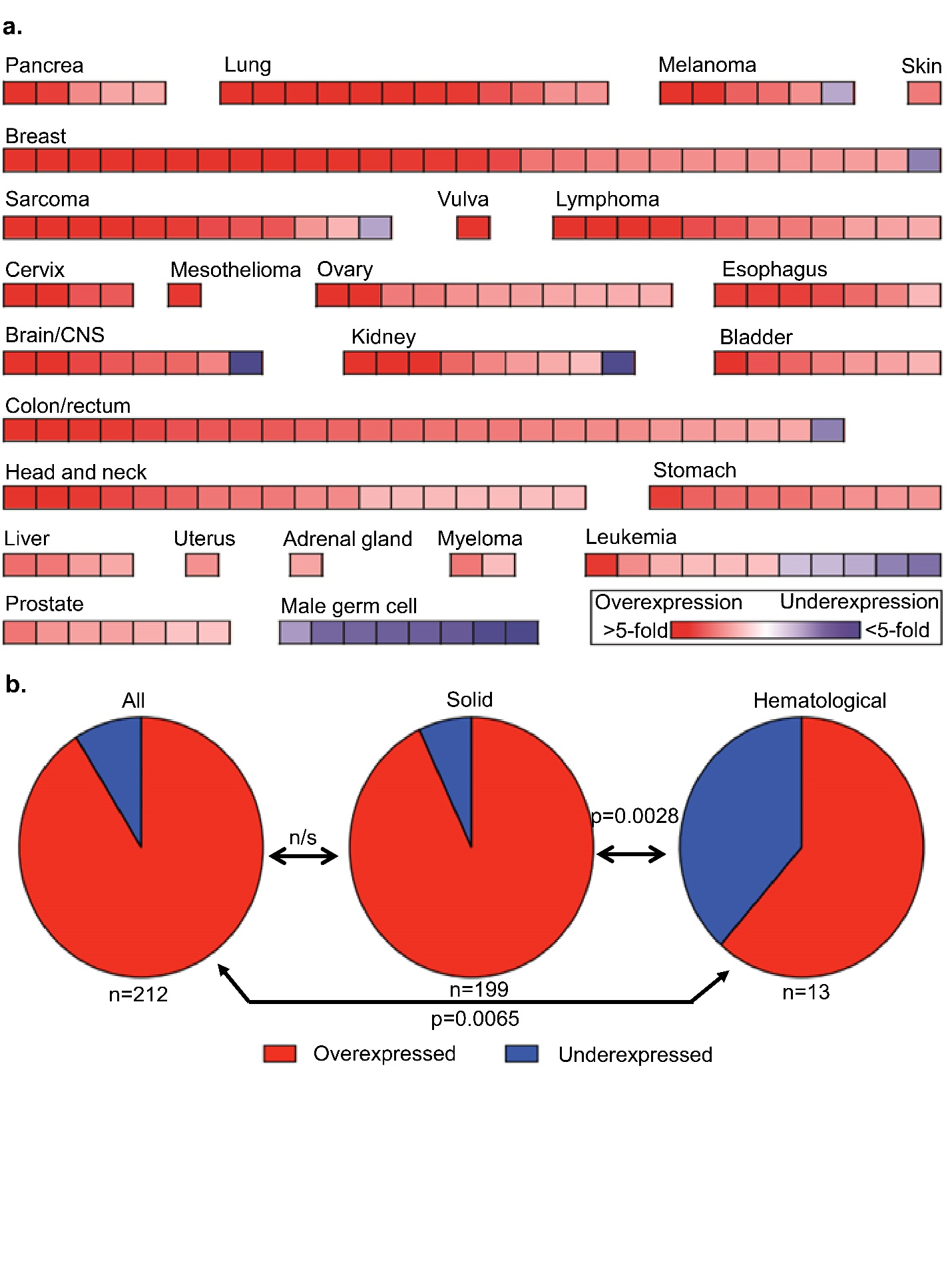


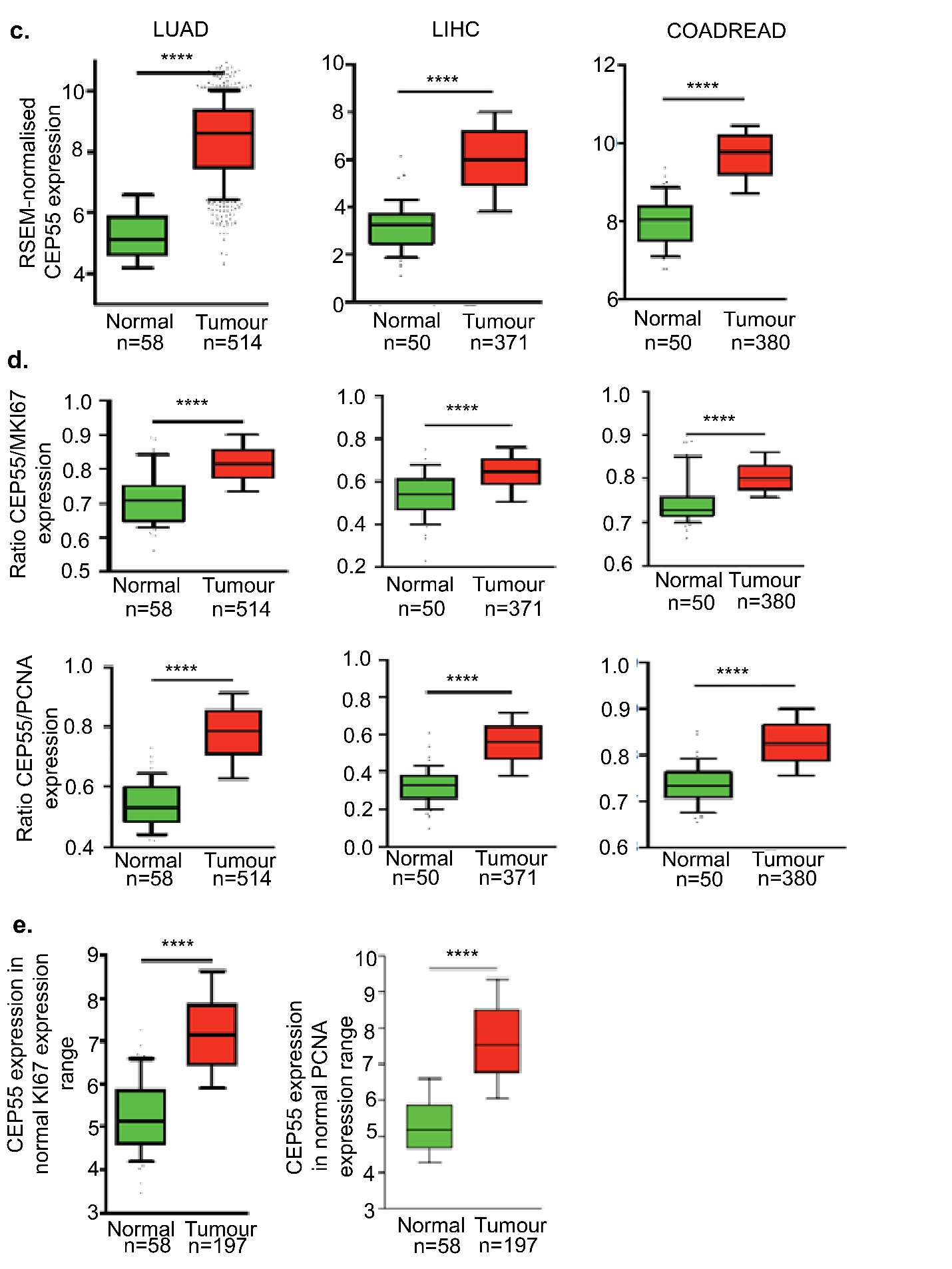


Supplementary Fig1: CEP55 is overexpressed in a broad range of cancers independent of a proliferation-associated effect.

(a) Gene expression of CEP55 in multiple data sets of various cancer types, analyzed using the Oncomine database^52^. Overall, 7403 tumor samples to 1467 normal control samples of matched tissue type were compared and we observed that the expression of CEP55 to be significantly higher than in matched normal tissue. A total of 212 data sets were identified showing statistically significant deregulated expression of CEP55 in tumours compared to matched normal control tissues. Each box represents a dataset. Studies showing significant CEP55 overexpression are shown in red, those showing significant underexpression in blue. Fold over- or under-expression is shown as indicated.

(b) Pie charts derived from the dataset described in (A) implying significant CEP55 expression differences compared to normal tissue (n=212) wherein 193 case studies (91%) illustrated significant overexpression, while 19 case studies (9%) showed significant underexpression. Proportion of studies showing significant overexpression (red) and underexpression (blue) is shown. Fisher’s exact tests was used to determine the respective *P-values*.

(c) RSEM-normalised gene expression of CEP55 levels, performed using the TCGA^53^ dataset, in lung adenocarcinoma (LUAD), liver hepatocellular carcinoma (LIHC) and or colorectal adenocarcinoma (COADREAD) samples compared to matched normal control samples. Collectively, the datasets illustrate that CEP55 is significantly upregulated in respective tumors in comparison to normal control tissue. Student t’ tests was used to determine *P-value;* <0.0001 (****).

(d) Ratios of CEP55/MKI67 expression (upper panel) and CEP55/PCNA (lower panel) RSEM-normalised expression levels in Lung adenocarcinoma (LUAD), Liver hepatocellular carcinoma (LIHC) and colorectal adenocarcinoma (COADREAD) of datasets as in (C).

(e) Representation of the tumors that express MKI67 at levels in the same range as normal samples show significantly elevated CEP55 expression levels than the normal samples. These data illustrate that expression of CEP55 is significantly higher in tumors than in normal tissue, even after compensation for the expression of the cell proliferation markers MKI67 (upper panel) or PCNA (lower panel), indicating that CEP55 expression in tumors is cell cycle-independent. Mann-Whitney t- test was used to determine *P-value;* <0.0001(****).


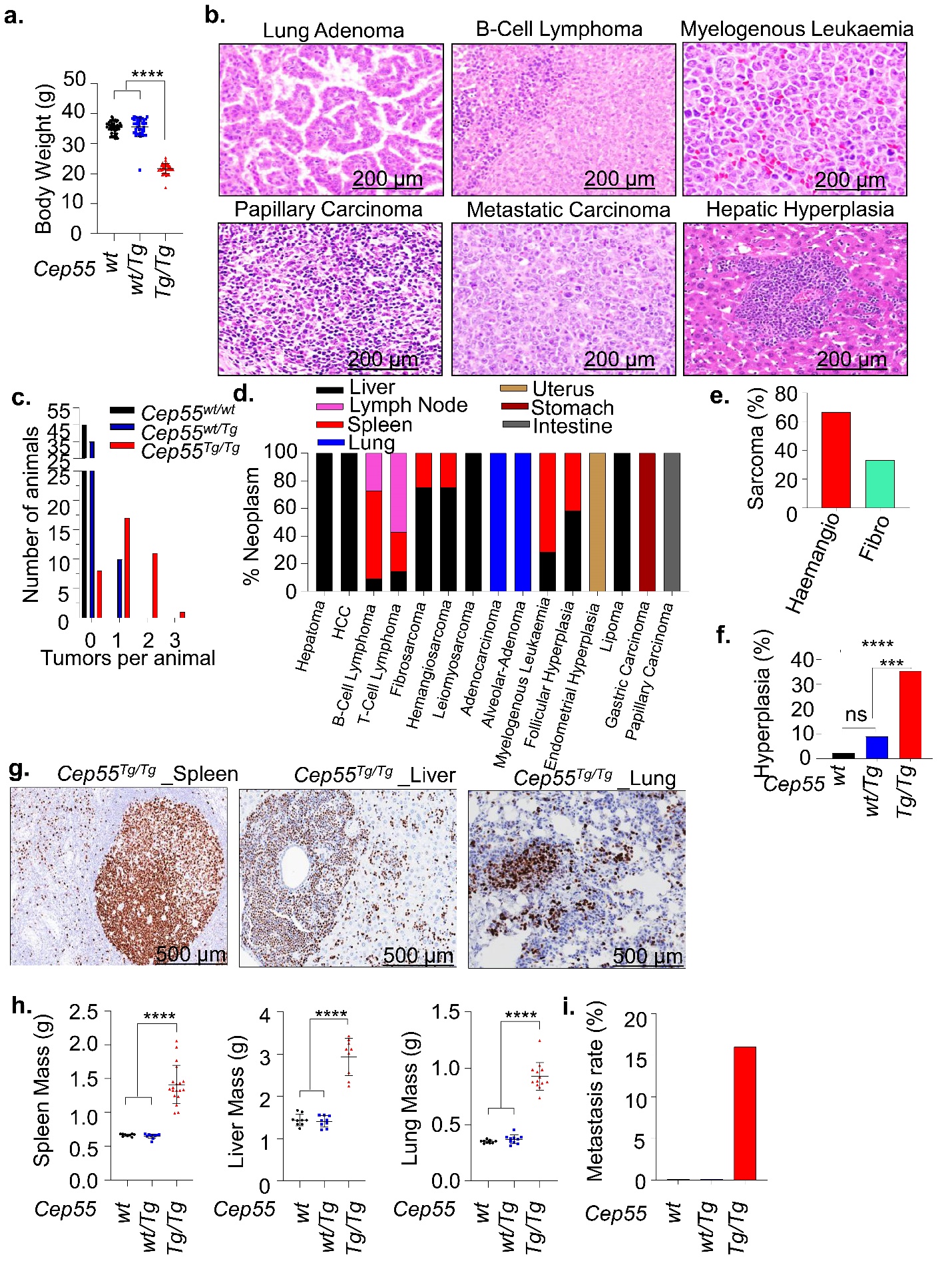


Supplementary Fig2: Spontaneous tumourigenesis induced by Cep55 overexpression in vivo.

(a) Body weight observed at the end of experimental cohorts evaluating survival of mice of each genotype (n>40 per group). Error bars represent the ± SD. One-way ANOVA test was performed to determine *P-value;* <0.0001 (****).

(b) Representation of H&E-stained microscopic images of indicated tumor lesions from different organs of tumors-bearing *Cep55^Tg/Tg^* mice; (scale bars, 200 µm).

(c-f) The overall distribution of indicated tumour lesions among indicated organs of tumour-bearing *Cep55^Tg/Tg^* mice (HCC=hepatocellular carcinoma). Percentage of tumor burden (c), types of organs that they originated from (d), Sarcoma (e) and hyperplasia (f) observed in tumour-bearing *Cep55^Tg/Tg^* mice. Error bars represent the ± SD. One-way ANOVA test was performed to determine *P-value*; <0.001 (***), <0.0001 (****) and ns (not significant ).

(g) Representative Ki67-stained microscopic images of indicated tumor lesions compared to surrounding near normal tissue from different organs of tumors-bearing *Cep55^Tg/Tg^* mice; (scale bars, 500 µm).

(h) Body weights of spleen, liver and lung from individual animal that harbor tumors. Error bars represent the ± SD. One-way ANOVA test was performed to determine P-value <0.0001 (****).

(i) Percentage of metastasis incidence observed in the tumor bearing *Cep55^Tg/Tg^* mice.


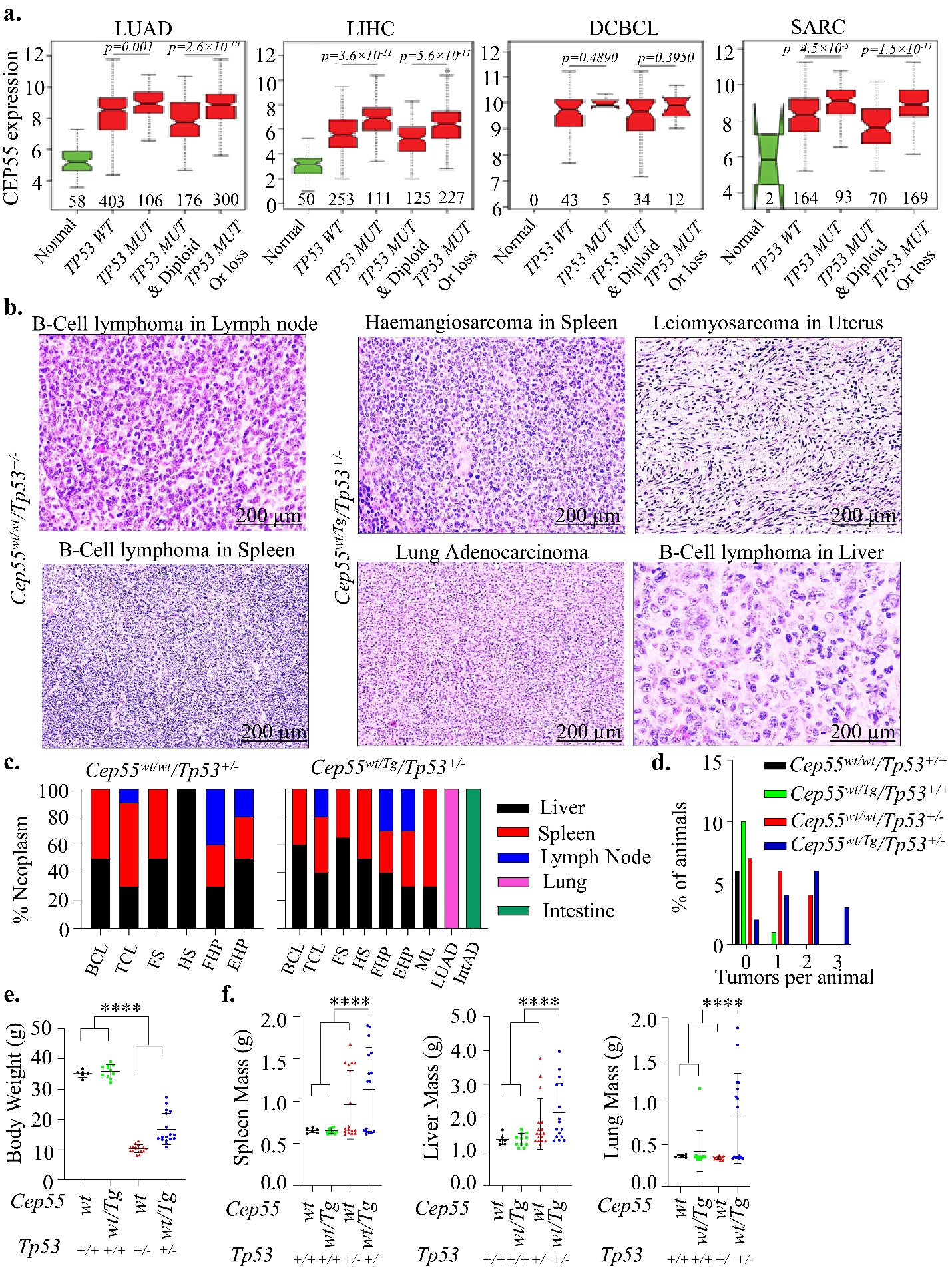


Supplementary Fig3: Loss of Trp53 leads to early tumor latency in Cep55 overexpressing mice.

(a) Boxplots showing CEP55 expression in indicated tumors and matched normal samples in lung adenocarcinoma (LUAD), liver hepatocellular carcinoma (LIHC), Diffuse large B-cell lymphoma (DLBCL), Sarcoma (SARC)) patients. TP53 copy number and/or mutation status is indicated (wherein *WT* indicates presence of *Wildtype* allele and *MUT* indicates presence of mutation). Numbers of samples for each column are shown above the x-axis. P-values were determined using Mann-Whitney t- test; ****p<0.0001.

(b) Representation of H&E stained microscopic images of selected sections of indicated tumors lesions from different organs of tumor-bearing mice of respective genotypes; (scale bars, 200 µm).

(c) Percentage of the overall distribution of indicated tumor lesions among indicated organs of tumor-bearing mice. Abbreviations: B-cell lymphoma (BCL), T-cell lymphoma (TCL), Fibrosarcoma (FS), Hemangiosarcoma (HS), Follicular hyperplasia (FHP), endometrial hyperplasia (EHP), yelogenous leukemia (ML), lung adenocarcinoma (LUAD), intestinal adenocarcinoma (IntAD).

(d) Percentage of mice of indicated genotype showing indicated number of tumors (tumour burden).

(e) Body weight of mice observed at the end of experiment for each genotype (n≥10 per group). Error bars represent the ± SD. One-way ANOVA test was performed to determine *P-value* <0.0001 (****).

(f) Statistical representation of the respective weights of spleen, liver and lung observed in the indicated genotypes. Error bars represent the ± SD from the entire experimental cohort. One-way ANOVA test was performed to determine *P-value;* <0.0001 (****).


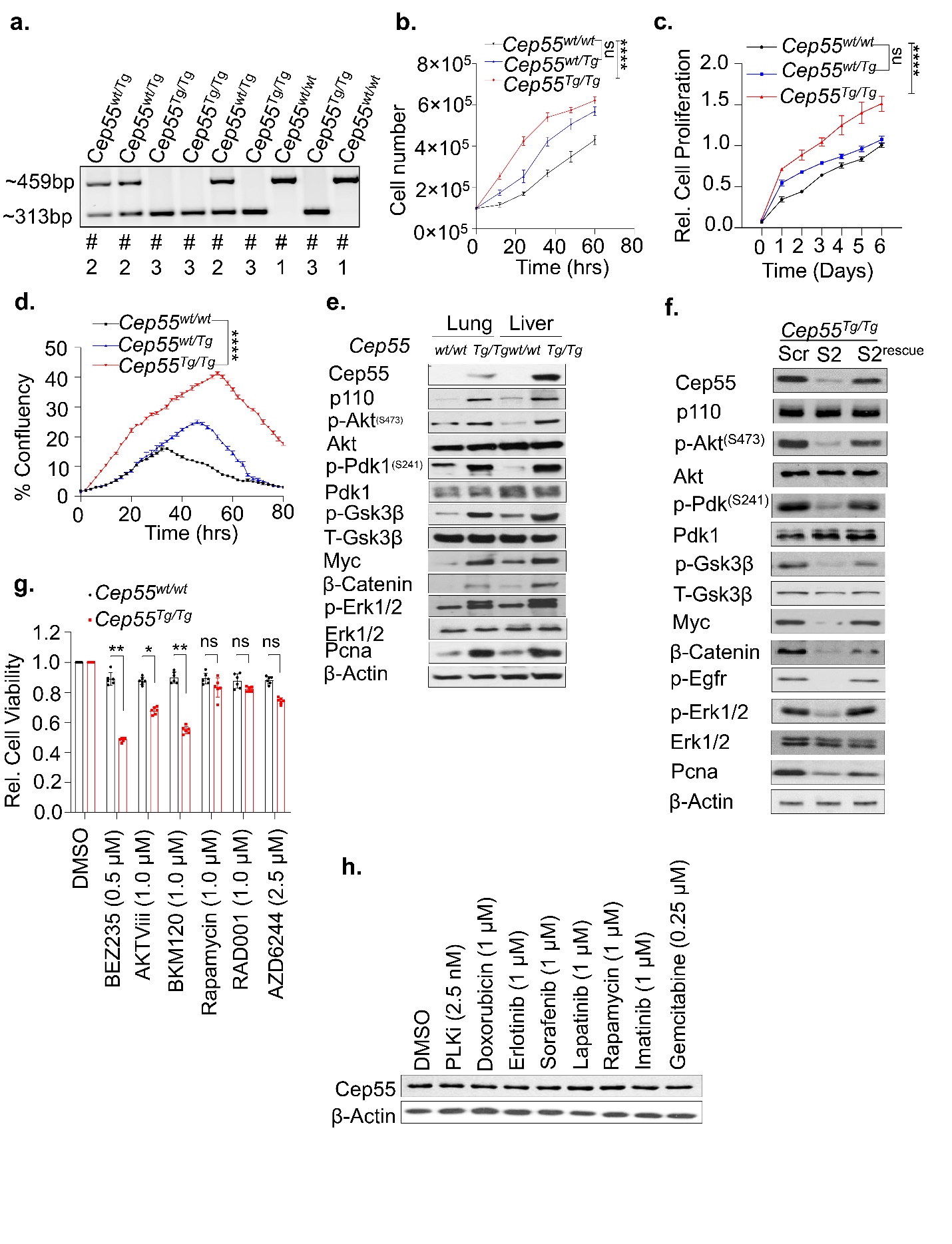


Supplementary Fig4: Cep55 overexpression promotes cell proliferation advantage in MEFs.

(a) Representation of genotyping of DNA isolated from the primary MEFs of each indicated genotype using PCR, showing the presence of amplicons of the expected size for each genotype. The amplicon size of the respective transgene allele has been indicated as (~459 bp) and the *wildtype* allele as (~313 bp). The numbers represents, #1: *Cep55^wt/wt^*, #2: *Cep55^wt/Tg^* and #3: *Cep55^Tg/Tg^*.

(b) Relative fold change in doubling time of immortalized MEFs from each genotype. Error bars represent the ± SD from two independent experiments. One-way ANOVA test was performed to determine *P-value* <0.0001(****).

(c) Cell viability of immortalized MEFs of each genotype, performed using MTS assay, measured per day over a period of 6 days. Error bars represent the ± SD from two independent experiments. One-way ANOVA test was performed to determine *P-value* <0.0001 (****).

(d) Cell proliferation observed in the immortalized MEFs of indicated genotype at different time points during serum starved conditions measured using Incucyte,. Error bars represent the ± SD from two independent experiments. One-way ANOVA test was performed to determine *P-value* <0.001 (****).

(e) Immunoblot analysis of indicated whole tissue lysates collected from six-month old littermates of respective genotypes of. β-Actin was used as loading control.

(f) Immunoblot analysis of the whole cell lysates collected from the immortalized *Cep55^Tg/Tg^* MEF’s with or without Cep55 loss and rescue of the phenotype by introducing exogenous Cep55 in Cep55-depleted cells. Cep55 was depleted in *Cep55^Tg/Tg^* MEFs using the *siRNA S2* (10 nM, targeting the *UTR* region) followed by transfection of cells with Cep55 expressing plasmid. β-Actin was used as a loading control.

(g) Cell viability of the immortalized MEFs of each genotype after 48hrs of treatment with indicated small molecule inhibitors treated as per the designated concentration. Error bars represent the ± SD from three independent experiments. Student's t‐test was performed to determine *P-value*; <0.05 (*) and <0.01 (**) and ns (not significant ).

(h) Immunoblot analysis of the whole cell lysates collected from the immortalized *Cep55^Tg/Tg^* MEF’s 24 hours after treatment with the respective anti-proliferation drugs. β-Actin was used as a loading control.


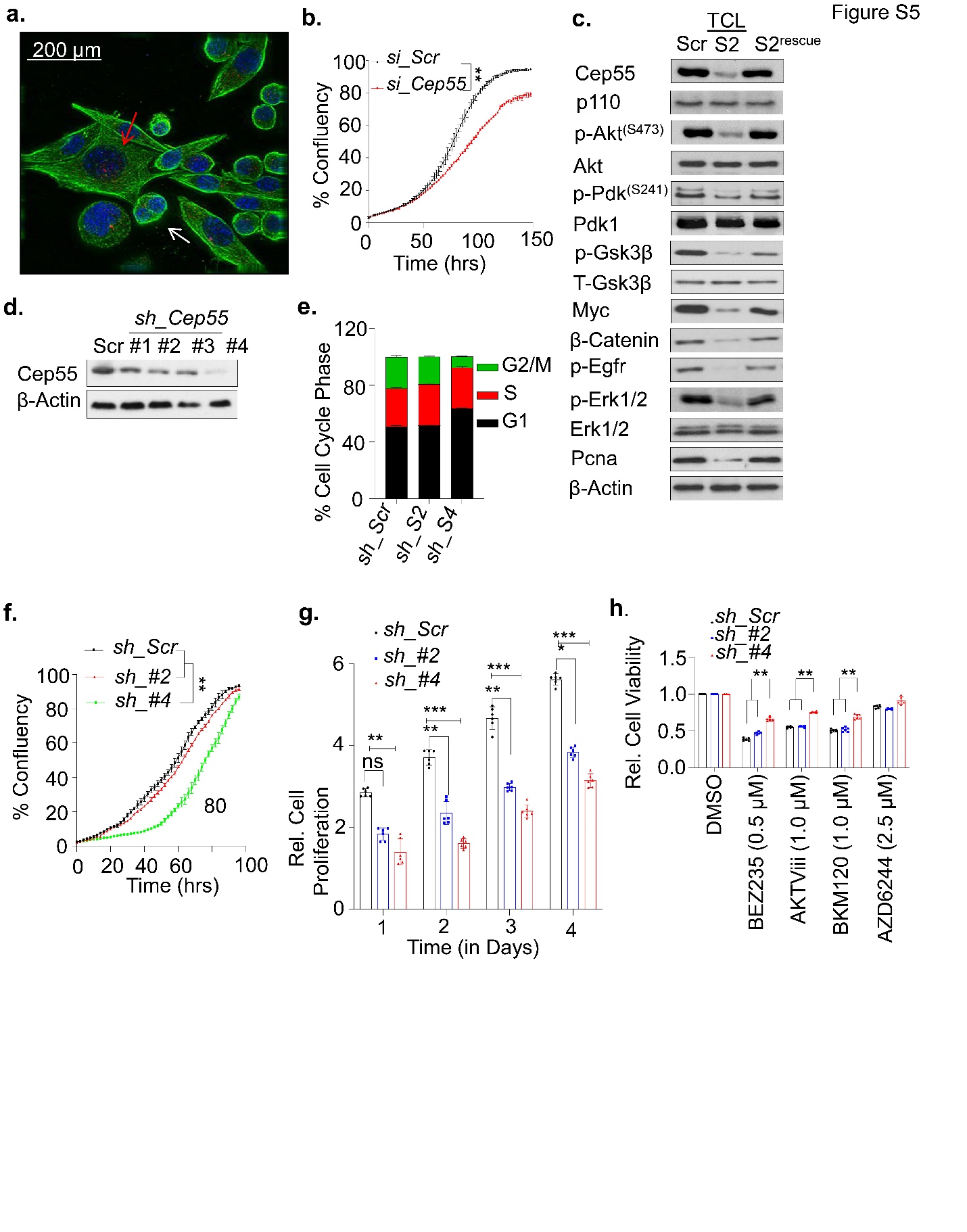
 Supplementary Fig5: Loss of Cep55 expression is associated with proliferation defect.

(a) Representative images of cell morphology of the TCLs isolated from the haemangiosarcoma found in (Fig1 Ci), stained with α-tubulin (green), γ-tubulin (red) and DAPI (blue). The red arrow indicated presence of multinucleated cells and the white arrow represents presence binucleated cells.

(b) Effect of Cep55 depletion using *siCep55* (10 nM) on cell proliferation, assessed using the IncuCyte ZOOM^®^ live-cell imager. The percentage of cell confluence was determined using an IncuCyte mask analyser (right panel). Error bars represent the ± SD from two independent experiments. Student t’ test was performed to determine *P-value;* <0.01 (**).

(c) Immunoblot analysis of the whole cell lysates collected from the TCL with or without Cep55-depletion and rescue of signaling defects by ectopic Cep55 expression. Cep55 was depleted in TCL using the *siRNA S2* (10 nM, targeting the *UTR* region) followed by transfection of cells with *Cep55* expressing plasmid. β-Actin was used as a loading control.

(d) Immunoblot analysis of whole-cell lysates of TCLs to validate extent of Cep55 depletion with indicated *shCep55* sequences (indicated as #S1- #S4 with *sh_Scr* as control) in the TCLs. β-Actin was used as loading control.

(e) Cell cycle profile of respective TCL clones determine using FACS. Error bars represent the ± SD from three independent experiments. Two-way ANOVA test was performed to determine *P-value* as demonstrated in Supplementary Table 3.

(f) Effect of Cep55 depletion on cell proliferation in TCLs assessed as described in (B). Error bars represent the ± SD from two independent experiments. Student t’ test was performed to determine *P-value* <0.01 (**).

(g) Relative cell proliferation of Cep55-depleted TCL compared to control (Scr) performed using MTS assay at the indicated time points. Error bars represent the ± SD from three independent experiments. One-way ANOVA test was performed to determine *P-value;* <0.05 (*),<0.01 (**), <0.001 (***) and ns (not significant ).

(h) Relative cell viability of the indicated TCL clones after 48hrs of treatment with indicated small molecule inhibitors treated as per the designated concentration. Error bars represent the ± SD from three independent experiments. One-way ANOVA test was performed to determine *P-value* , <0.05 (*) and <0.01 (**).


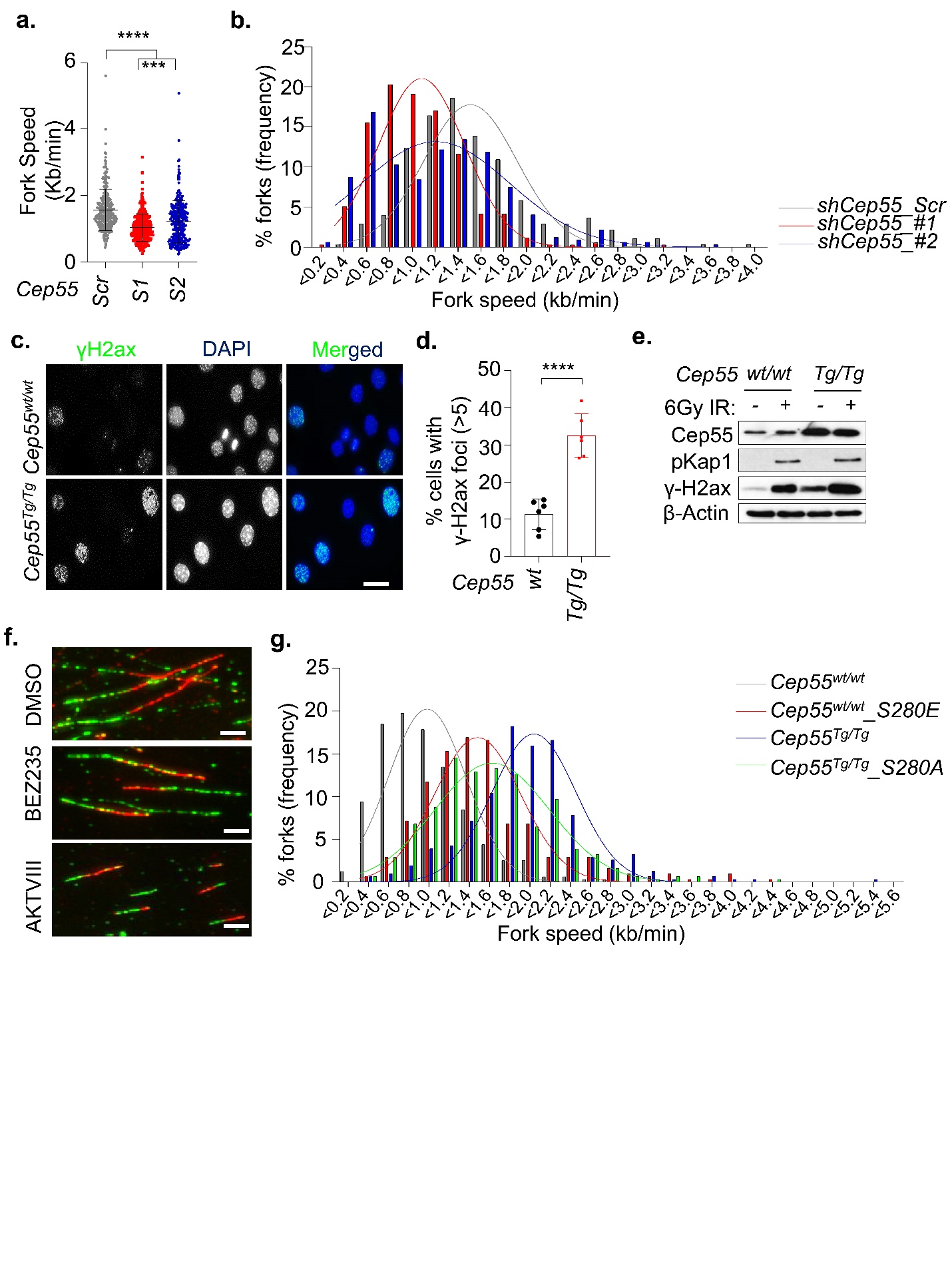
 Supplementary Fig6: Cep55 overexpression causes replication stress.

(a, b) Statistical representation of velocity of progressing forks (a) and distributions of replication fork speeds frequency (b) was determined using DNA fiber analysis upon *Cep55* knockdown in the immortalized *Cep55^Tg/Tg^* MEFs. At least 300 fibers from each cell line were analysed from two independent experiments with error bars representing the ±SD for panel A. Unpaired t test with and without Welch's correction between two groups was used to determine the statistical *P-value* <0.001 (***), <0.0001 (****).

(c) Representative images of immunofluorescence (left panel) demonstrating presence of DNA double strand break marked by γ-H2ax (green) observed in indicated genotypes (Scale bar, 100 μm).

(d) Statistical representation showing percentage of γ-H2ax positive cells (>5 foci of γ-H2ax /cell) in the immortalized MEFs of indicated genotypes MEFs (right panel). Error bars represent the ± SD from three independent experiments. Student's t‐test was performed to determine *P-value*; <0.0001 (****).

(e) Immunoblot analysis of indicated proteins in cell extracts obtained from respective immortalized MEFs after challenged with 6-Gy irradiation. β-actin was used as a loading control.

(f) Representative images of the DNA fibres obtained from immortalized *Cep55^Tg/Tg^* MEFs after the indicated treatments with Pi3k inhibitors. Cells were treated with the inhibitors for 6 hours before DNA labelling was performed.

(g) Statistical representation of velocity of progressing forks of the MEFs of each genotypes that were transiently transfected with 1.5µg of indicated mutant constructs respectively (*CHK1* mutants -*S280A* and *S280E* respectively) for 24 hours as presented in Figure 4H.


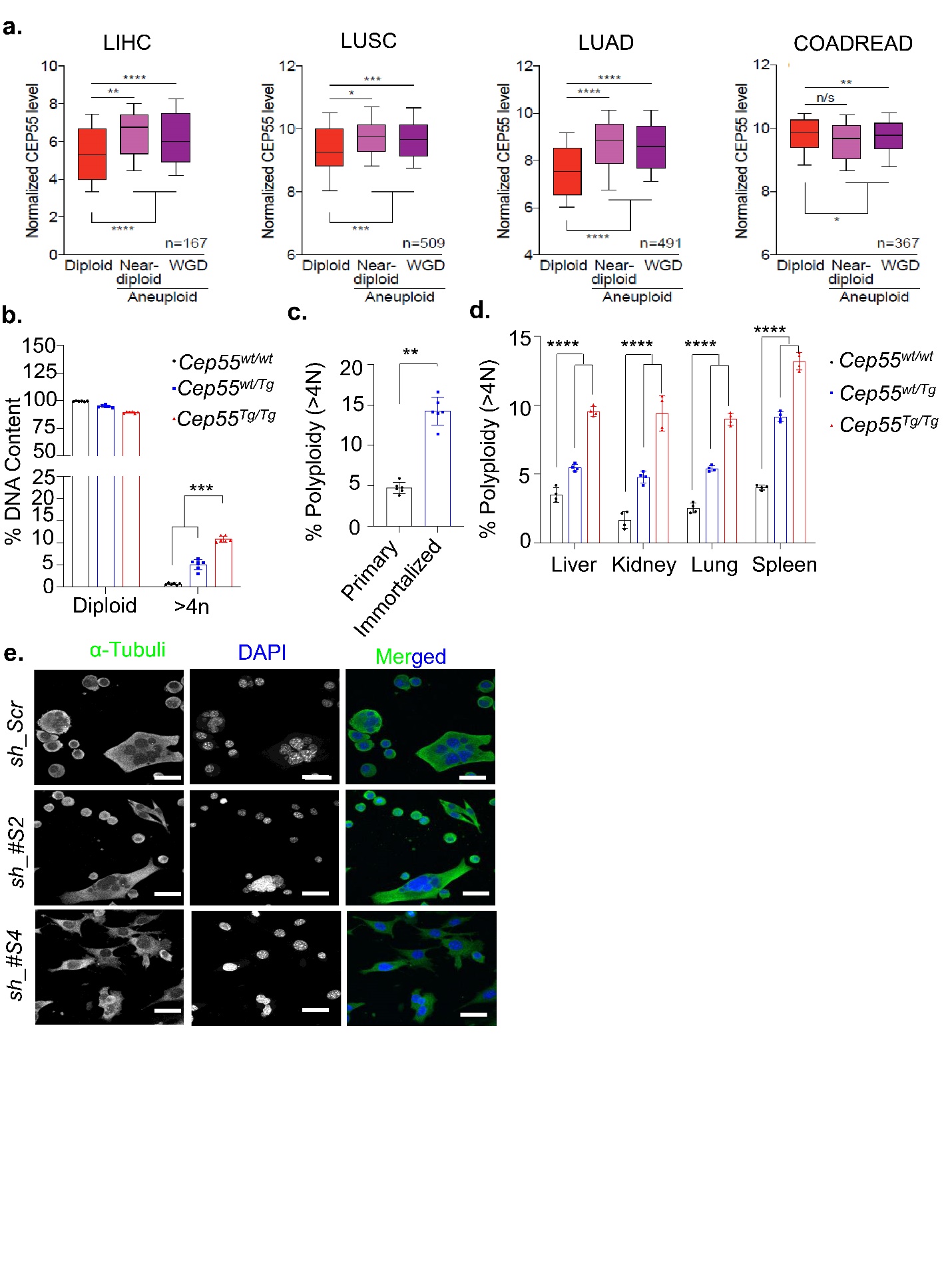


Supplementary Fig7: Cep55 overexpression causes genomic instability.

(a) Boxplots showing CEP55 expression in tumors whose genomes are diploid, near-diploid aneuploid or aneuploid after whole-genome doubling (WGD). Data are from the TCGA liver hepatocellular carcinoma (LIHC), lung squamous cell carcinoma (LUSC), lung adenocarcinoma (LUAD) and colorectal adenocarcinoma (COADREAD) datasets. Mann-Whitney U tests was used to determine *P-value* <0.05 (*), <0.01 (**), <0.001 (***), <0.0001 (****).

(b, c) Polyploidy analysis (>4N DNA content) determined using FACS. Error bars represent the ± SD from three independent experiments with two replicates. One-way ANOVA test was performed to determine *P-value;* <0.001 (***).

(d) Percentage polyploidy observed in the indicated tissues of age-matched mice of each genotypes. Error bars represent the ± SD from 4 mice per group. One-way ANOVA test was performed to determine *P-value;* <0.0001 (****).

(e) Immunofluorescence showing genomic instability observed among the respective *shCep55*-depleted isogenic TCLs clones as indicated by the presence of multiple nuclei (marked by DAPI staining) compared to control counterpart. The entire cell (cytoplasm) was marked by α-tubulin (green), while the centrosomes were marked by γ-tubulin (red) (Scale bar, 100 μm).


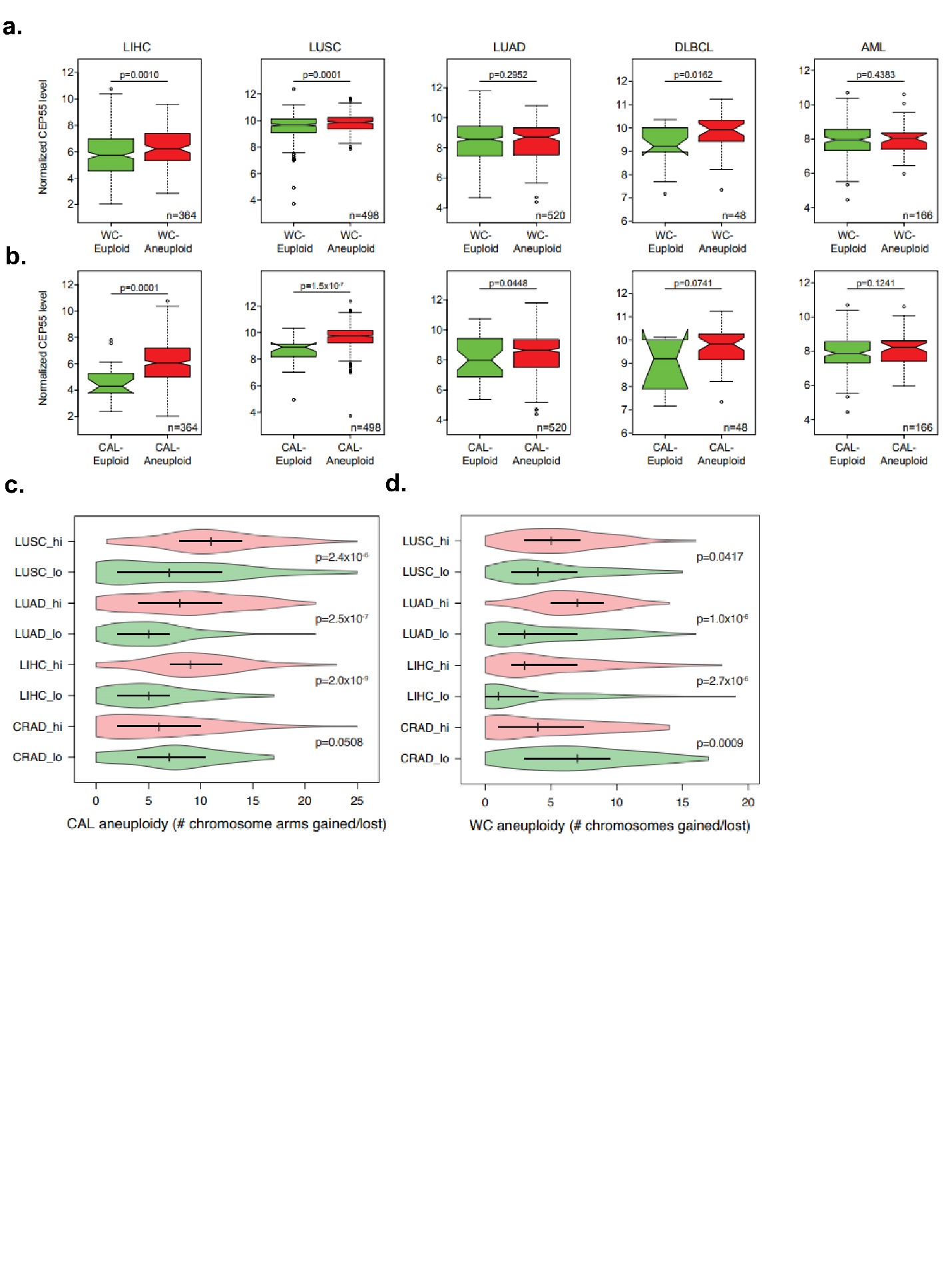
 Supplementary Fig8: Association of CEP55 overexpression with aneuploidy.

(a) Boxplots representation showing CEP55 expression in indicated tumors with whole-chromosome (WC)-euploid and WC-aneuploid genomes. The data was defined using the TCGA  Liver Hepatocellular Carcinoma (LIHC),  Lung Squamous Cell Carcinoma (LUSC), and  Lung Adenocarcinoma (LUAD) datasets (described in Supplementary Fig5A)^50^.

(b) Boxplots as in (A) but at the chromosome arm level (CAL).

(c) Boxplots representation demonstrating the chromosome arm-level (CAL) aneuploidy, i.e., total number of chromosome arms gained or lost per sample, with respect to the highest (hi) and lowest (lo) CEP55 mRNA expression quartiles from TCGA RNAseq data.

(d) Boxplots representation demonstrating the whole-chromosome (WC) aneuploidy, i.e., the total number of whole chromosomes gained or lost per sample, with respect to the highest (hi) and lowest (lo) CEP55 mRNA expression quartiles from TCGA RNAseq data. For all of the above, Mann-Whitney U tests was used to determine *P-value*.


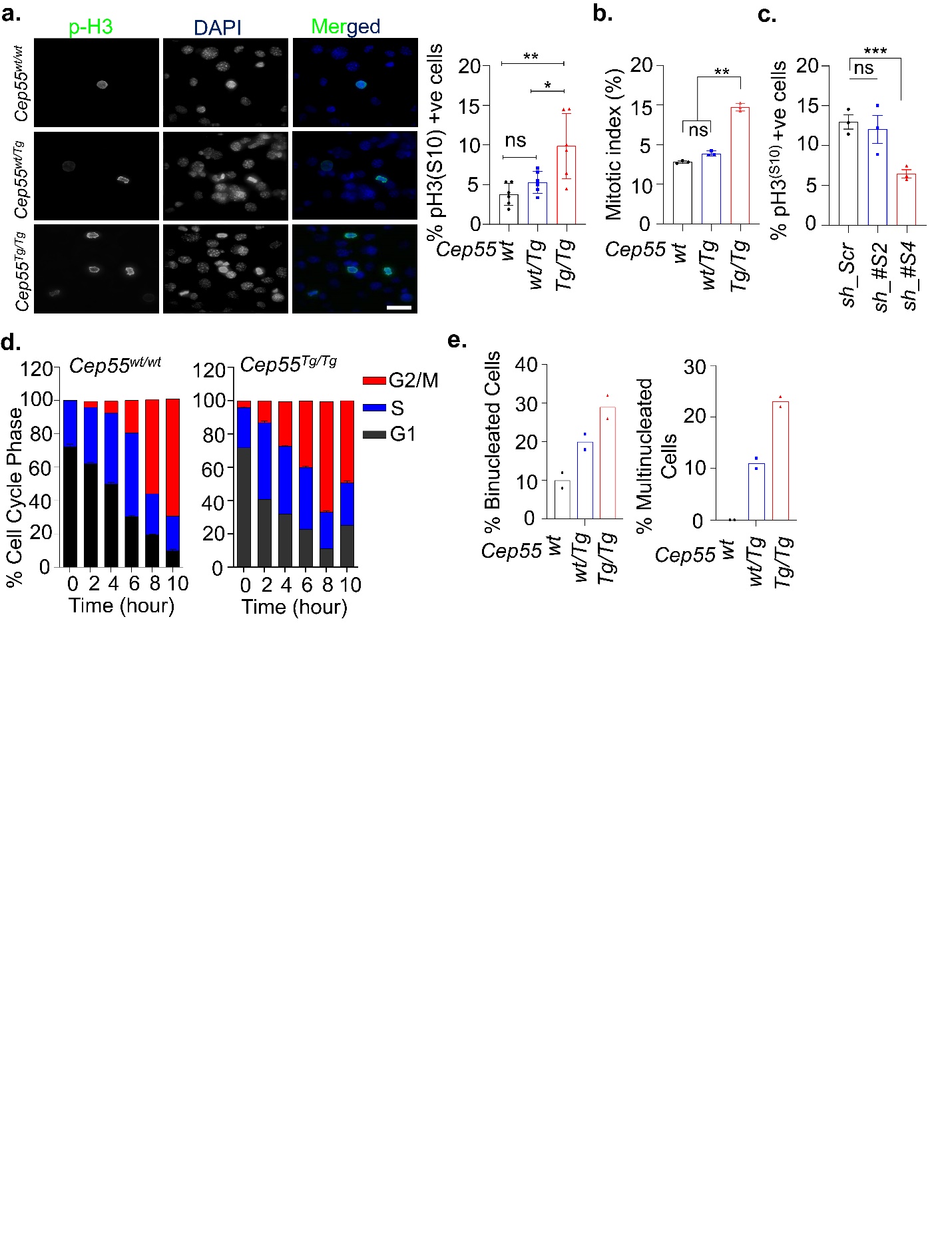


Supplementary Fig9: Mitotic cell fate in Cep55 overexpressing MEFs.

(a) Representative images of immunofluorescence demonstrating mitotically active cells observed in immortalized Cep55^Tg/Tg^ MEFs as compared to other counterparts. Mitotic cells are marked by phospho-histone H3 (green) and the nucleus is marked by DAPI (blue). Scale bar, 100 μm (left panel). Statistical representation of phospho-histone H3^+ve^ cells in the MEFs of indicated genotypes (right panel). Error bars represent the ± SD from three independent experiments. One-way ANOVA test was performed to determine P-value; 0.05 (*), <0.01 (**) and ns (not significant).

(b) Mitotic index (number of rounded cells/overall cells in an area) observed in the immortalized MEFs of indicated genotypes using bright field Olympus Xcellence IX81 time-lapse microscopy per-field. Overall, 300 cells were counted (~40 cells per field) of each genotype. Error bars represent the ± SD from three independent experiments. Student's t‐test was performed to determine P-value; <0.01 (**) and not significant (ns).

(c) Percentage of phospho-histone H3^+ve^ cells observed in the indicated *shCep55* depleted isogenic clones quantified as in A. Error bars represent the ± SD from three independent experiments. One-way ANOVA test was performed to determine P-value; <0.001 (***) and not significant (ns).

(d) Cell cycles profiles of immortalized MEFs of indicated genotypes determined by propidium iodide staining followed by FACS analysis. The cells were first synchronized by double-thymidine block and released in regular culture media following which, they were collected after 2-hour intervals.

(e) Boxplots showing the percentage of binucleated (left panel) and multinucleated cells (right panel) of indicated MEFs calculated using time-lapse microscopy as shown in Figure 6A.


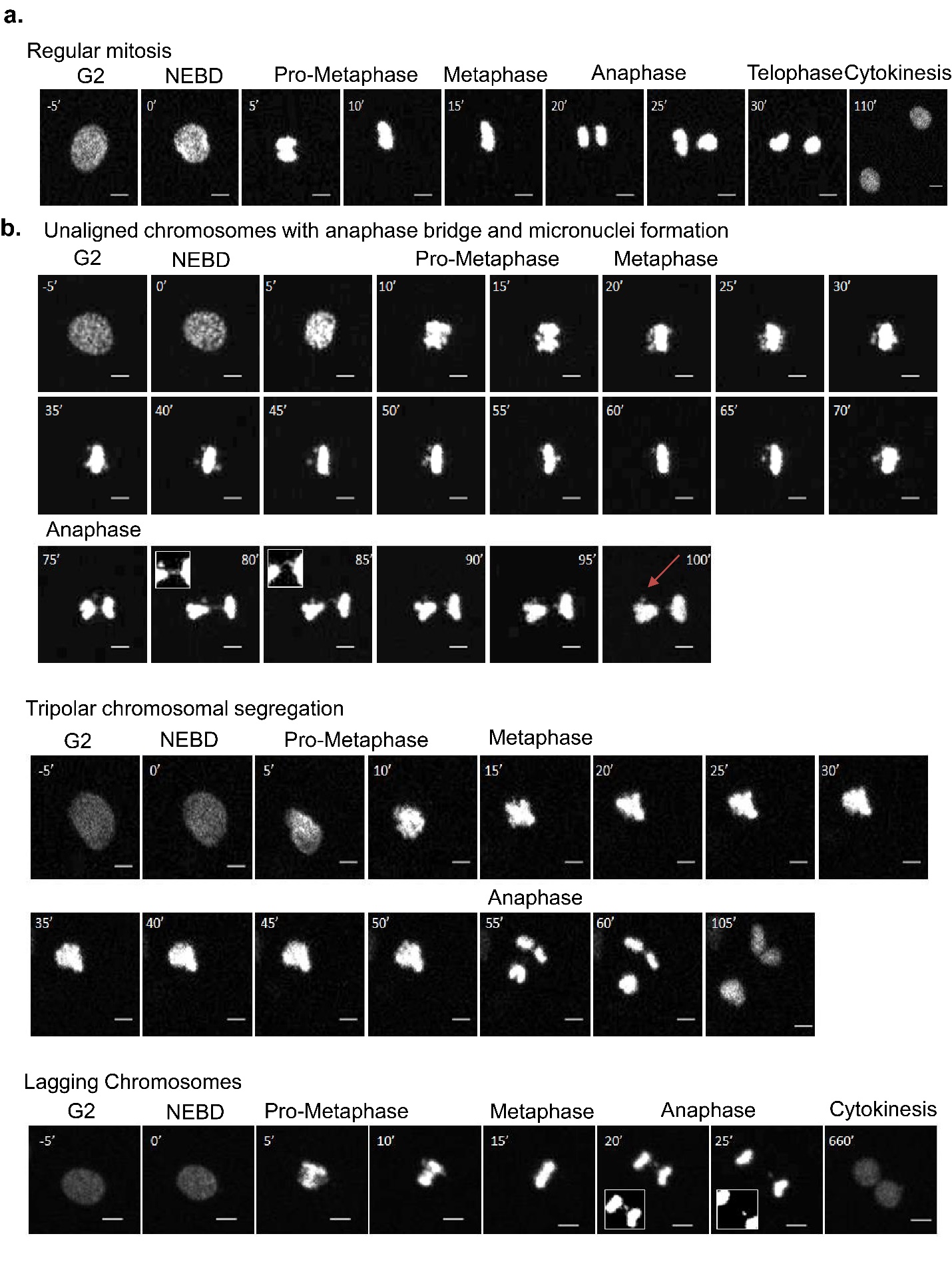


Supplementary Fig10: Cep55 overexpression causes mitotic defects.

(a, b) Representative images showing normal (a) and perturbed mitoses (b). Individual cells of immortalized Cep55^Tg/Tg^ MEFs were tracked using bright-field Olympus Xcellence IX81 time-lapse microscopy and mitotic anomalies were determined (Scale bar, 100 μm). Red arrow indicates the formation of micronuclei at the end of Anaphase.


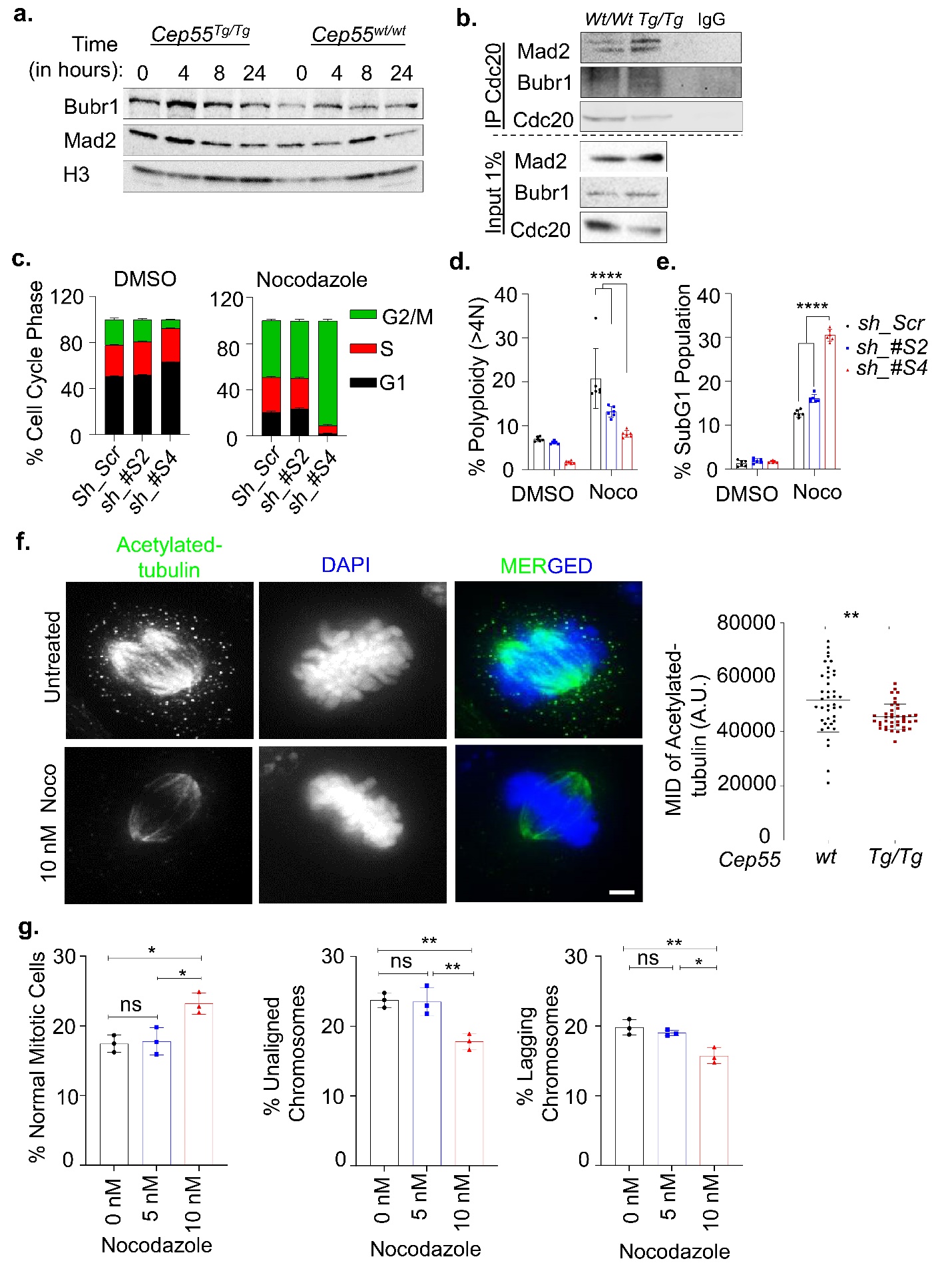


Supplementary Fig11: Cep55 overexpression and its impact on microtubule stability.

(a) Immunoblot analysis of the SAC activation from whole cell lysates collected at the indicated time point from the immortalized MEFs of indicated genotype post synchronization using double thymidine block and released in nocodazole (0.5 μM). Total histone H3 was used as loading control.

(b) Co-immunoprecipitation was performed on double thymidine blocked cells which were released into nocodazole for four hours and cell extract were immunoprecipitated with Cdc20 antibody and immunoblotted with indicated antibodies. 1% of total lysate was used as inputs control. As a specificity control, rabbit IgG was also used.

(c) Cell cycle profile of the respective *shCep55* depleted isogenic clones in the presence or absence of nocodazole (0.5 μM) determined using FACS***.*** Error bars represent the ± SD from three independent experiments. Two-way ANOVA test was performed to determine *P-value* as demonstrated in Supplementary Table 3.

(d,e) Percentage of polyploidy (>4N DNA contents) and SubG1 population determined using FACS in the respective shCep55 depleted isogenic clones in presence or absence of nocodazole (0.5 μM). Error bars represent the ± SD from three e independent experiments with two replicates each. One-way ANOVA test was performed to determine *P-value* <0.0001 (****).

(f) Representative images of acetylated tubulin (green) of metaphase stages of immortalized *Cep55^Tg/Tg^* MEFs post treatment with nocodazole (10 nM) (Scale bar, 100 μm) (left).Statistical representation of the mean integrated density (MID) of acetylated tubulin observed post nocodazole (10 nM) treatment among immortalized *Cep55^Tg/Tg^* MEFs (right). The intensity was calculated using Image J software wherein n=20 metaphase cells were calculated per genotype. Error bars represent the ± SD from two independent experiments. Student's t‐test was performed to determine P-value <0.01 (**).

(g) Percentage of the mitotic defects (unaligned metaphase plates and lagging chromosomes) alongside regular mitosis observed in the immortalized *Cep55^Tg/Tg^* MEFs post nocodazole treatment (5 nM and 10 nM). Error bars represent the ± SD from three independent experiments. Student's t‐test was performed to determine *P-value* <0.05 (*), <0.01 (**) and not significant (ns).


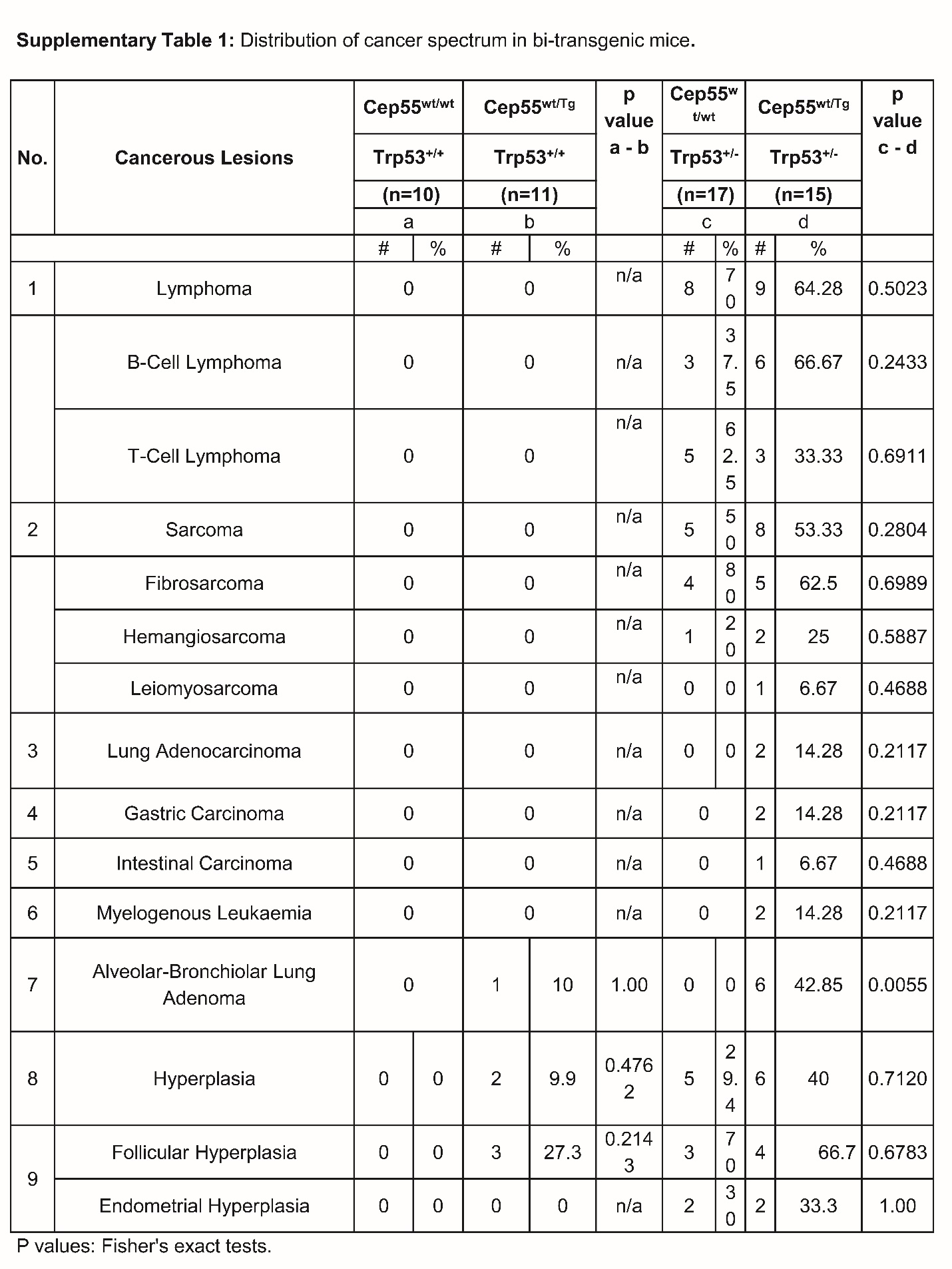


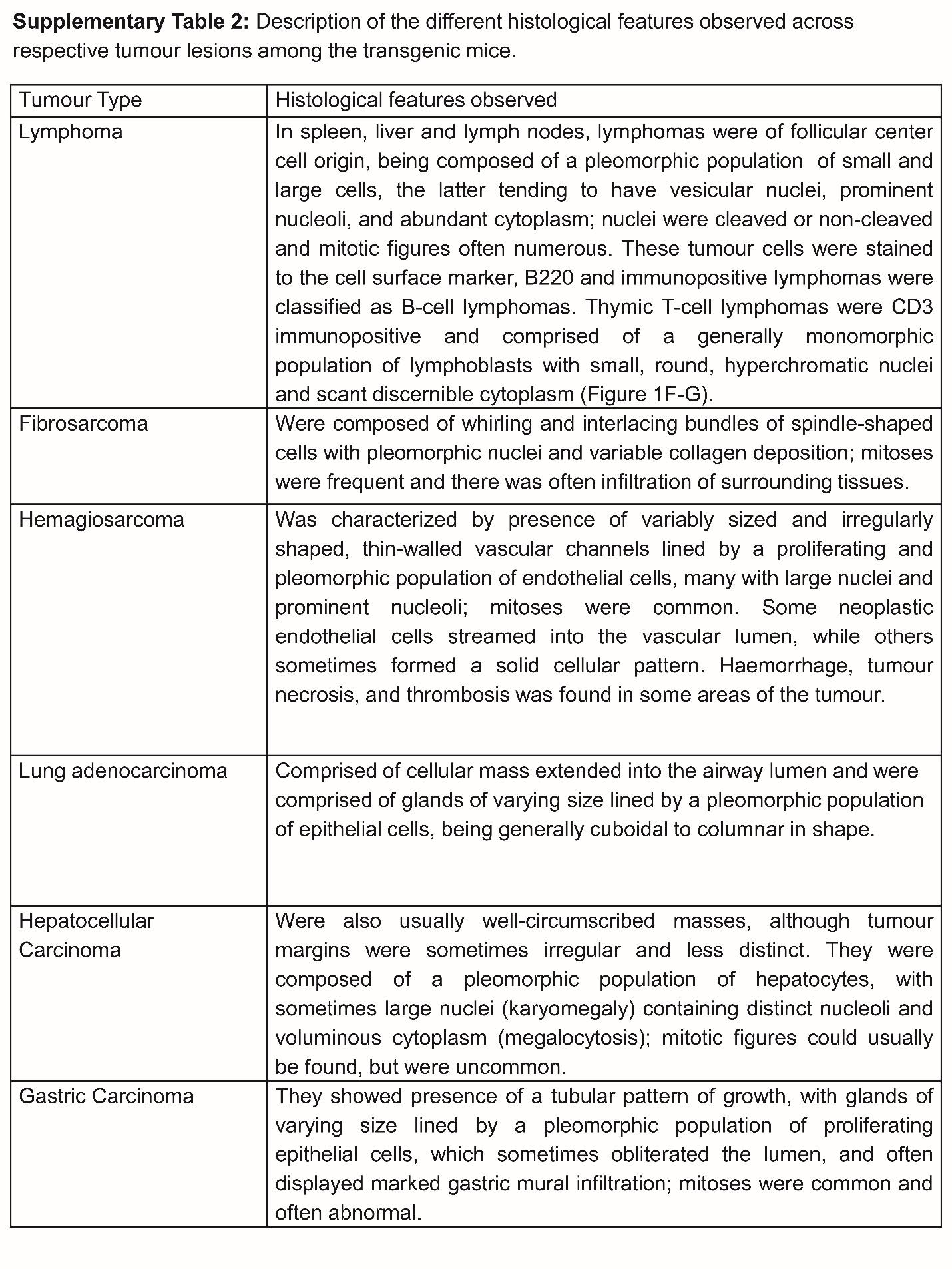


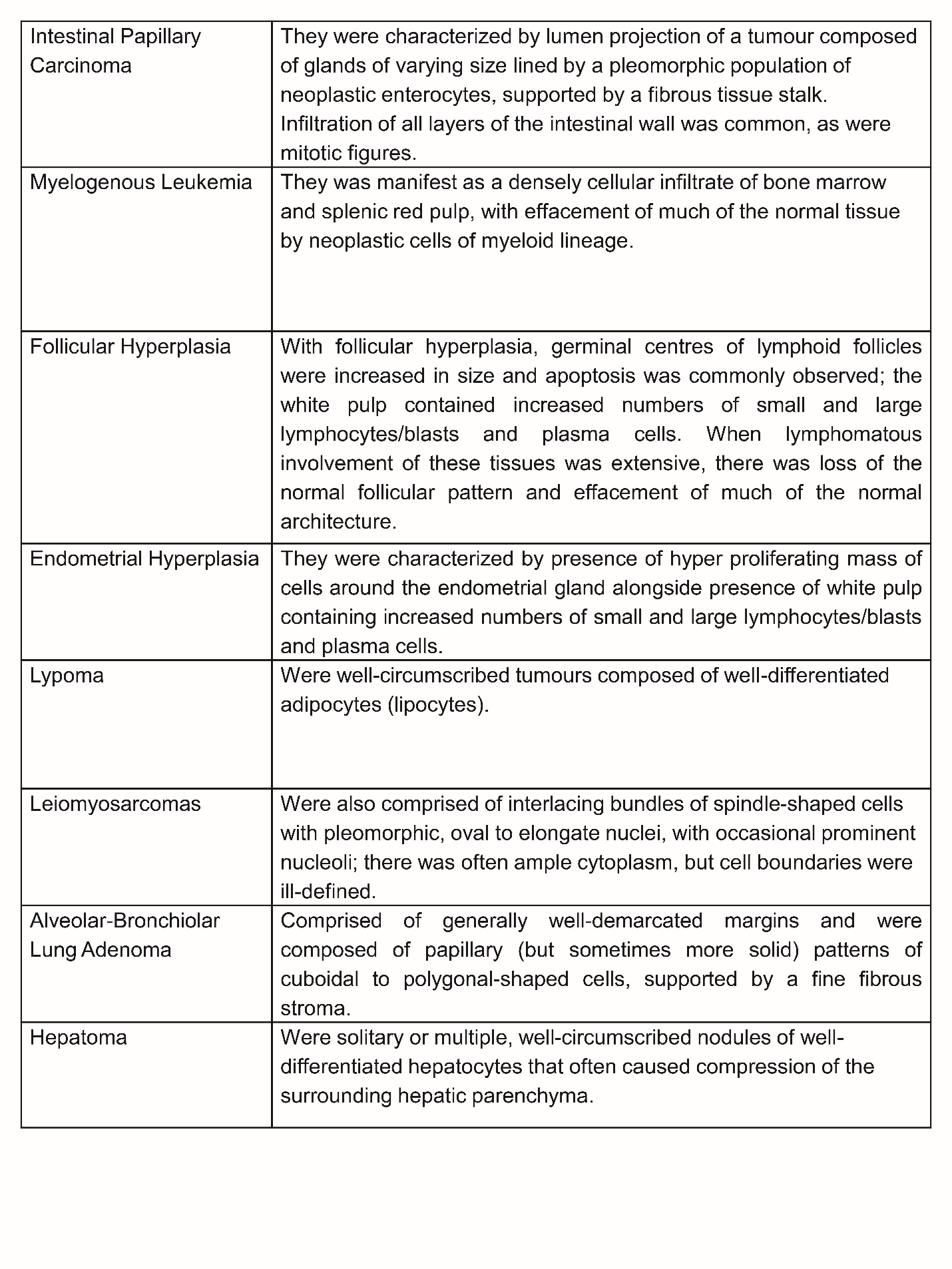


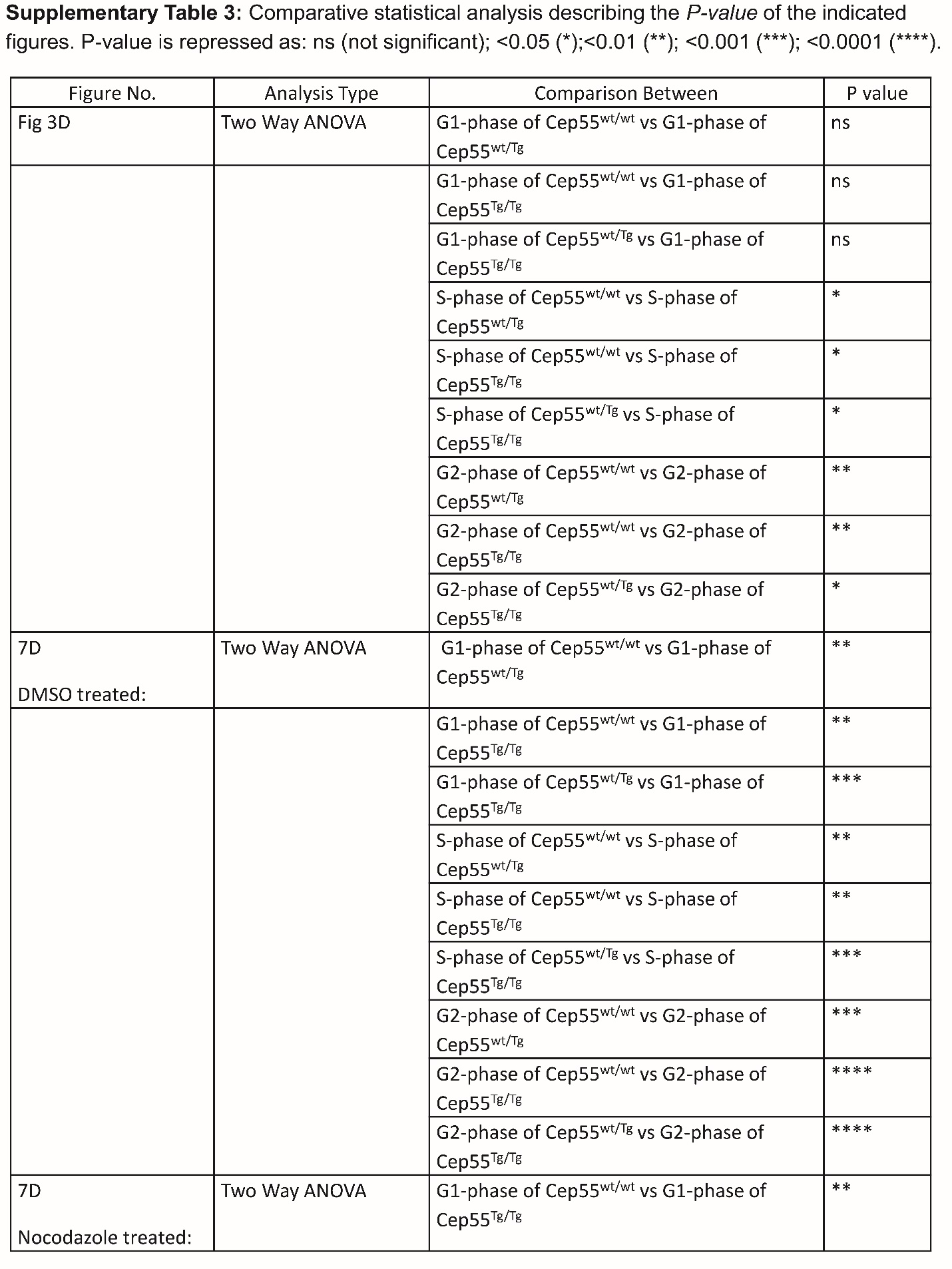


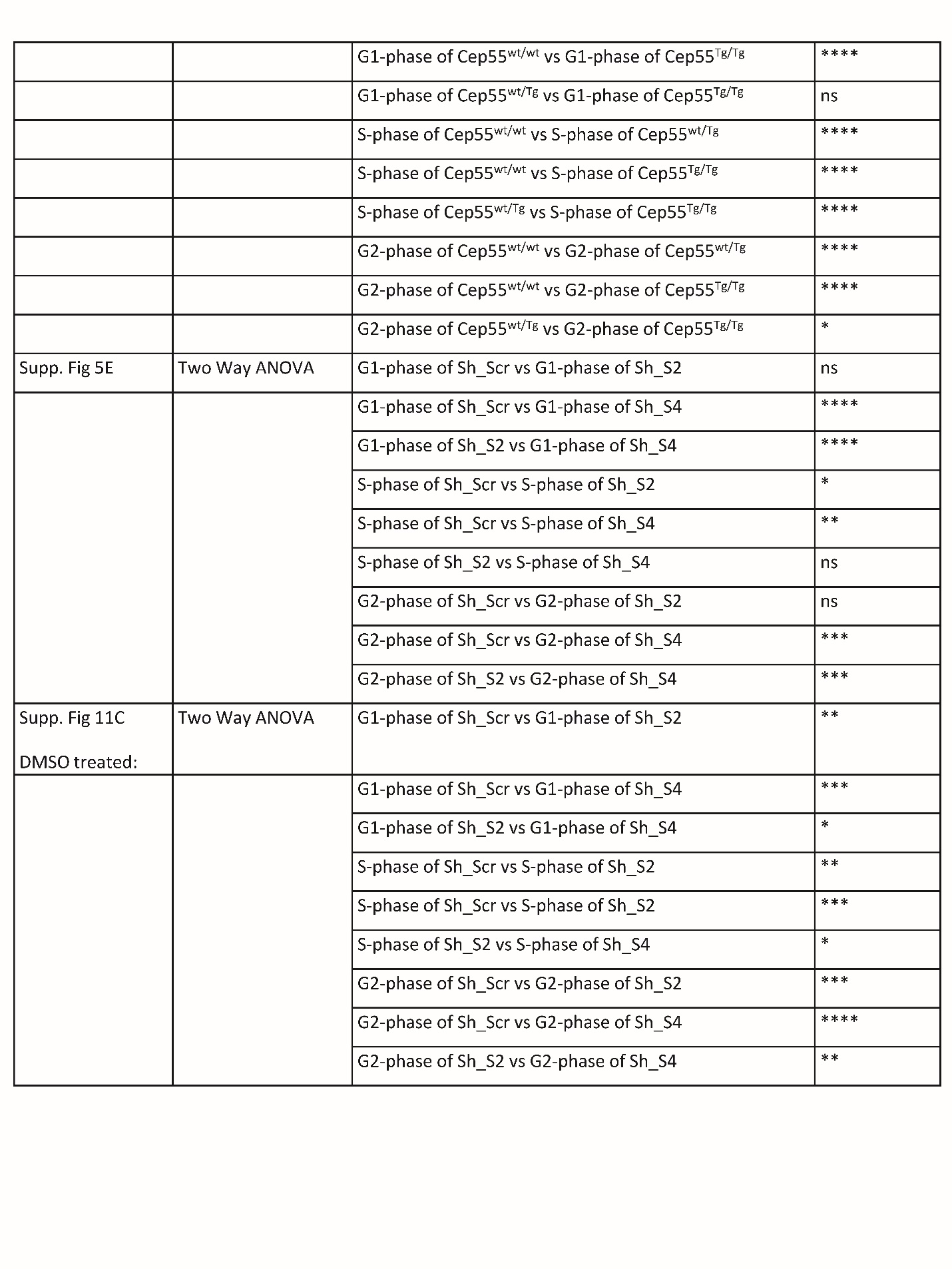


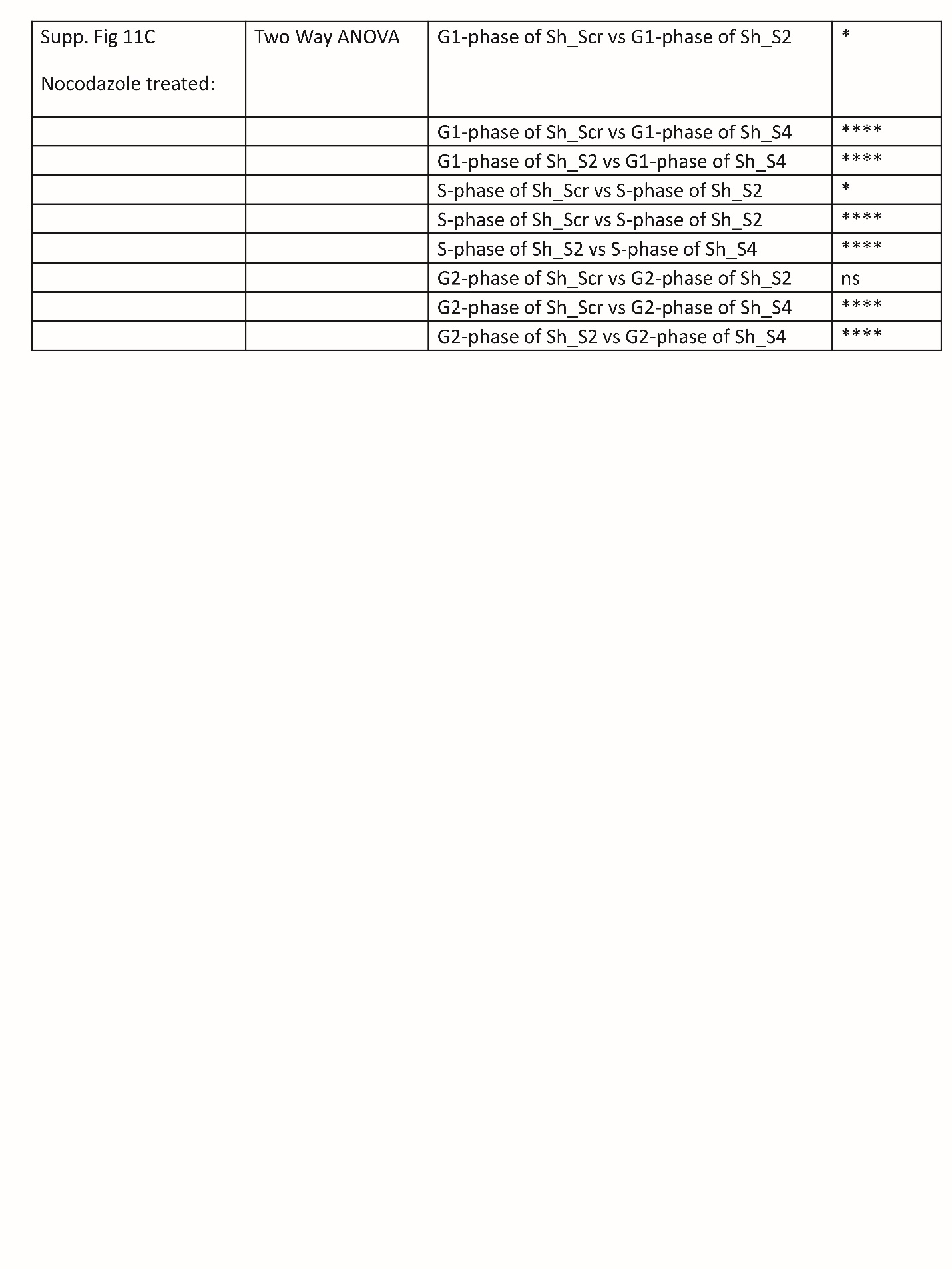

Supplement: Supplementary file 1 — Supplementary Information [file 42003_2020_1304_MOESM1_ESM.docx]
