# Supplementary material for: Cep55 overexpression promotes genomic instability and tumorigenesis in mice
Source: Commun Biol. 2020 Oct 21;3:593. doi: 10.1038/s42003-020-01304-6 (PMC7578791; doi:10.1038/s42003-020-01304-6)
Supplement: Supplementary file 2 — Description of Additional Supplementary Files [file 42003_2020_1304_MOESM2_ESM.pdf]

Description of Additional Supplementary Files

File Name: Supplementary Data 1
Description: Source data for all main figures.
