# Supplementary material for: Cep55 overexpression promotes genomic instability and tumorigenesis in mice
Source: Commun Biol. 2020 Oct 21;3:593. doi: 10.1038/s42003-020-01304-6 (PMC7578791; doi:10.1038/s42003-020-01304-6)
Supplement: Supplementary file 4 — Reporting Summary [file 42003_2020_1304_MOESM4_ESM.pdf]

nature research

Corresponding author(s): Murugan Kalimutho

Last updated by author(s): 2020/09/11

Reporting Summary

Nature Research wishes to improve the reproducibility of the work that we publish. This form provides structure for consistency and transparency in reporting. For further information on Nature Research policies, see Authors & Referees and the Editorial Policy Checklist .

Statistics

For all statistical analyses, confirm that the following items are present in the figure legend, table legend, main text, or Methods section.

n/a	Confirmed	
☐	☒	The exact sample size (*n*) for each experimental group/condition, given as a discrete number and unit of measurement
☐	☒	A statement on whether measurements were taken from distinct samples or whether the same sample was measured repeatedly
☐	☒	The statistical test(s) used AND whether they are one- or two-sided<br>*Only common tests should be described solely by name; describe more complex techniques in the Methods section.*
☐	☒	A description of all covariates tested
☐	☒	A description of any assumptions or corrections, such as tests of normality and adjustment for multiple comparisons
☐	☒	A full description of the statistical parameters including central tendency (e.g. means) or other basic estimates (e.g. regression coefficient) AND variation (e.g. standard deviation) or associated estimates of uncertainty (e.g. confidence intervals)
☒	☐	For null hypothesis testing, the test statistic (e.g. *F*, *t*, *r*) with confidence intervals, effect sizes, degrees of freedom and *P* value noted<br>*Give P values as exact values whenever suitable.*
☒	☐	For Bayesian analysis, information on the choice of priors and Markov chain Monte Carlo settings
☒	☐	For hierarchical and complex designs, identification of the appropriate level for tests and full reporting of outcomes
☒	☐	Estimates of effect sizes (e.g. Cohen's *d*, Pearson's *r*), indicating how they were calculated

Our web collection on statistics for biologists contains articles on many of the points above.

Software and code

Policy information about availability of computer code

Data collection	N/A
Data analysis	Graphpad Prism 7.02

For manuscripts utilizing custom algorithms or software that are central to the research but not yet described in published literature, software must be made available to editors/reviewers. We strongly encourage code deposition in a community repository (e.g. GitHub). See the Nature Research guidelines for submitting code & software for further information.

Data

Policy information about availability of data

All manuscripts must include a data availability statement. This statement should provide the following information, where applicable:

- Accession codes, unique identifiers, or web links for publicly available datasets
- A list of figures that have associated raw data
- A description of any restrictions on data availability

All data used in this study is provided in the form of figures. Raw data is also submitted as supplementary data file and available upon request.

Field-specific reporting

Please select the one below that is the best fit for your research. If you are not sure, read the appropriate sections before making your selection.

☒ Life sciences ☐ Behavioural & social sciences ☐ Ecological, evolutionary & environmental sciences

For a reference copy of the document with all sections, see nature.com/documents/nr-reporting-summary-flat.pdf

Life sciences study design

All studies must disclose on these points even when the disclosure is negative.

Sample size	Most of the experiments were repeated at least twice with duplicate or triplicate of technical replicates.
Data exclusions	No data is excluded from analysis unless otherwise stated in figure legends
Replication	Repeated 2-3 times.
Randomization	N/A
Blinding	N/A

Reporting for specific materials, systems and methods

We require information from authors about some types of materials, experimental systems and methods used in many studies. Here, indicate whether each material, system or method listed is relevant to your study. If you are not sure if a list item applies to your research, read the appropriate section before selecting a response.

Materials & experimental systems

n/a	Involved in the study
☐	☒ Antibodies
☐	☒ Eukaryotic cell lines
☐	☐ Palaeontology
☐	☒ Animals and other organisms
☐	☐ Human research participants
☐	☐ Clinical data

Methods

n/a	Involved in the study
☐	☐ ChIP-seq
☐	☒ Flow cytometry
☐	☐ MRI-based neuroimaging

Antibodies

Antibodies used	γ-H2ax (1:1000 dilution) S139 (05-636); Cell Signaling antibodies: PARP (#9542), pAKTS473 (#4060), AKT (#9272), pPdk1S241 (#3061), Pdk1(#3062) Chk1 (2G1D5) (#2360), p-GSK-3β(Ser9) (#9336), GSK-3β (#9315), p-Histone H3 (#9706) (1:1000 dilution); Millipore antibody: Chk2 (1:500 dilution) (Clone 7) (05-649); BD Pharmingen antibody: β-actin (1:2000 dilution) (612656); Bethyl antibody:pKap1(S824) (1:1000 dilution) (A300-767A). Immunoprecipitation was performed as per our previous publication45. Immunodetections were performed using Bubr1 (ab4637), Mad2 (CST4636S), CDC20 (CST14866A),γ-H2ax S139 (05-636; Millipore), p-Histone H3 (#9706; CST), α-Tubulin (T9026), γ-Tubulin (T5192), Acetylated tubulin (T7451; Sigma) and detyrosinated tubulin (ab48389; Abcam), Ki67 1:500 (anti-rabbit, Novacastra #NCL-ki67p), B220 1:500 (anti-rat, ThermoFischer Scientific #14-0452-82), CD3 1:250 (anti-rabbit, Abcam #ab5690), p21 1:500 (anti-rabbit, Abcam #ab188224), p53 1:400 (anti-rabbit, Abcam #ab131442).
Validation	Routinely used commercial antibodies and have been reported in our previous articles or in commercial websites

Eukaryotic cell lines

Policy information about cell lines

Cell line source(s)	MEF and Tumour cell lines were derived from mice embryos and tumours respectively using published protocols as described in the material and methods.
Authentication	N/A
Mycoplasma contamination	Check regularly for mycoplasma contamination.
Commonly misidentified lines (See ICLAC register)	N/A

Palaeontology

Specimen provenance	All animal work was approved by the QIMR Berghofer Medical Research Institute, Animal Ethics Committee (number A0707-606M) and was performed in strict accordance with the Australian code for the care and use of animals for scientific purposes.

Specimen deposition	N/A
Dating methods	N/A

☐ Tick this box to confirm that the raw and calibrated dates are available in the paper or in Supplementary Information.

Animals and other organisms

Policy information about studies involving animals; ARRIVE guidelines recommended for reporting animal research

Laboratory animals	Mixed strains of BALB/C and C57BL/6J.
Wild animals	N/A
Field-collected samples	Housing in QIMR Berghofer Medical Research Institute animal facility.
Ethics oversight	QIMR Berghofer Medical Research Institute animal ethics committee.

Note that full information on the approval of the study protocol must also be provided in the manuscript.

Human research participants

Policy information about studies involving human research participants

Population characteristics	N/A
Recruitment	N/A
Ethics oversight	N/A

Note that full information on the approval of the study protocol must also be provided in the manuscript.

Clinical data

Policy information about clinical studies

All manuscripts should comply with the ICMJEguidelines for publication of clinical research and a completedCONSORT checklist must be included with all submissions.

Clinical trial registration	N/A
Study protocol	N/A
Data collection	N/A
Outcomes	N/A

ChIP-seq

Data deposition

☐ Confirm that both raw and final processed data have been deposited in a public database such as GEO.

☐ Confirm that you have deposited or provided access to graph files (e.g. BED files) for the called peaks.

Data access links<br>*May remain private before publication.*	N/A
Files in database submission	N/A
Genome browser session<br>(e.g. UCSC)	N/A

Methodology

Replicates	N/A
Sequencing depth	N/A
Antibodies	N/A

Peak calling parameters | N/A

Data quality | N/A

Software | *Describe the software used to collect and analyze the ChIP-seq data. For custom code that has been deposited into a community repository, provide accession details.*

Flow Cytometry

Plots

Confirm that:

- [ ] The axis labels state the marker and fluorochrome used (e.g. CD4-FITC).
- [x] The axis scales are clearly visible. Include numbers along axes only for bottom left plot of group (a 'group' is an analysis of identical markers).
- [x] All plots are contour plots with outliers or pseudocolor plots.
- [x] A numerical value for number of cells or percentage (with statistics) is provided.

Methodology

Sample preparation	Cell lines were used.
Instrument	BD FACS CANTO A Machine
Software	ModFiT LT Version 4.0
Cell population abundance	Single Cell population was gated for the overall population. Cell cycle was analyed using PI staining.
Gating strategy	Simple gating strategy was utilised, with overall population being gated for FSC-A vs SSC-A, following which single cells were gated to FSC-H vs FSC-A. The single cell population was then gated to PI positivity using histogram.

- [ ] Tick this box to confirm that a figure exemplifying the gating strategy is provided in the Supplementary Information.

Magnetic resonance imaging

Experimental design

Design type	*Indicate task or resting state; event-related or block design.*
Design specifications	*Specify the number of blocks, trials or experimental units per session and/or subject, and specify the length of each trial or block (if trials are blocked) and interval between trials.*
Behavioral performance measures	*State number and/or type of variables recorded (e.g. correct button press, response time) and what statistics were used to establish that the subjects were performing the task as expected (e.g. mean, range, and/or standard deviation across subjects).*

Acquisition

Imaging type(s)	*Specify: functional, structural, diffusion, perfusion.*
Field strength	*Specify in Tesla*
Sequence & imaging parameters	*Specify the pulse sequence type (gradient echo, spin echo, etc.), imaging type (EPI, spiral, etc.), field of view, matrix size, slice thickness, orientation and TE/TR/flip angle.*
Area of acquisition	*State whether a whole brain scan was used OR define the area of acquisition, describing how the region was determined.*

Diffusion MRI ☐ Used ☐ Not used

Preprocessing

Preprocessing software	*Provide detail on software version and revision number and on specific parameters (model/functions, brain extraction, segmentation, smoothing kernel size, etc.).*
Normalization	*If data were normalized/standardized, describe the approach(es): specify linear or non-linear and define image types used for transformation OR indicate that data were not normalized and explain rationale for lack of normalization.*

Normalization template	*Describe the template used for normalization/transformation, specifying subject space or group standardized space (e.g. original Talairach, MNI305, ICBM152) OR indicate that the data were not normalized.*
Noise and artifact removal	*Describe your procedure(s) for artifact and structured noise removal, specifying motion parameters, tissue signals and physiological signals (heart rate, respiration).*
Volume censoring	*Define your software and/or method and criteria for volume censoring, and state the extent of such censoring.*

Statistical modeling & inference

Model type and settings	N/A
Effect(s) tested	N/A

Specify type of analysis: ☐ Whole brain ☐ ROI-based ☐ Both

Statistic type for inference (See Eklund et al. 2016)	N/A
Correction	N/A

Models & analysis

n/a	Involved in the study
☐	☐ Functional and/or effective connectivity
☐	☒ Graph analysis
☐	☐ Multivariate modeling or predictive analysis

Functional and/or effective connectivity	*Report the measures of dependence used and the model details (e.g. Pearson correlation, partial correlation, mutual information).*
Graph analysis	Graph analysis was performed using Graphpad Prism 7.02
Multivariate modeling and predictive analysis	*Specify independent variables, features extraction and dimension reduction, model, training and evaluation metrics.*
